# Privacy-Preserving Federated Learning for Artistic Image Classification in Visual IoT Sensor Networks

**DOI:** 10.3390/s26175654

**Published:** 2026-09-05

**Authors:** Shuyi Wang, Baoping Wang

**Affiliations:** 1 Faculty of International Tourism and Management, City University of Macau, Macao, China; t25091100749@cityu.edu.mo; 2 School of Management, Guangdong University of Science and Technology, Dongguan 523070, China

**Keywords:** visual Internet of things, artistic image classification, federated learning, differential privacy, non-IID data, edge intelligence, communication-efficient learning

## Abstract

Visual Internet-of-Things (IoT) cameras and institution-controlled edge gateways increasingly collect artwork images in museums, galleries, and heritage sites. Centralizing these images can expose collection contents, exhibition layouts, and contextual information. This paper proposes FedArtSense, a privacy-preserving federated learning framework for artistic style and medium classification. FedArtSense combines discrepancy-adaptive dual-space prototype alignment, client-level differential privacy for model and prototype releases, and importance-aware shared sparsification compatible with secure aggregation. Experiments on WikiArt, ArtBench-10, and a seven-class Behance Artistic Media subset use emulated non-IID client partitions, persistent acquisition shifts, constrained uplinks, and client dropout. Under the default client-level target $\left(\epsilon ,\delta \right)=\left(6,10^{-5}\right)$, FedArtSense obtains accuracies of 64.2%, 81.2%, and 75.3%, respectively, while reducing cumulative WikiArt uplink traffic to 11.5 GiB. The results support FedArtSense as a privacy–utility–communication trade-off for gateway-assisted artistic image classification; retrieval, detection, aesthetic prediction, and direct battery-powered camera training are outside the evaluated scope.

## 1. Introduction

Networked cameras and institution-controlled edge gateways are increasingly used to digitize, monitor, and organize artworks in museums, galleries, restoration laboratories, cultural heritage sites, and public exhibition spaces. Unlike a conventional centralized image collection, a visual Internet-of-Things (IoT) deployment produces images at geographically and administratively distributed sites. This study focuses on artistic style and medium classification; collection retrieval, object detection, and aesthetic prediction are not evaluated. Transferring sensor images to a central server can reveal private collection contents, unpublished artworks, restoration progress, ownership information, exhibition arrangements, or contextual details surrounding the artwork. The resulting problem is collaborative classification without surrendering control of local observations.

Federated learning offers a natural foundation for this objective because clients exchange learning information instead of raw data [1]. Nevertheless, data locality alone does not constitute a formal privacy guarantee. Model updates can encode information about the samples and clients that produced them, motivating client-level differential privacy [2]. The problem becomes particularly difficult for artistic images because privacy noise must be applied in a learning environment that is already statistically heterogeneous. Historical collections, contemporary design portfolios, and sensor-acquired exhibition images differ in artistic movement, visual medium, subject matter, illumination, viewpoint, color response, and compression artifacts. Research on computational style recognition has established that artistic imagery differs substantially from ordinary photography [3], while the Behance Artistic Media dataset demonstrates the breadth of contemporary media and depiction domains [4]. Consequently, a global model trained by direct averaging can suffer severe client drift even before privacy perturbation is introduced.

Existing federated optimizers address parts of this problem. Proximal regularization limits local parameter deviation under non-IID data [5], control variates correct biased local updates [6], and contrastive objectives improve agreement among client representations [7]. Prototype-based federation exchanges class-level feature summaries and is well suited to semantically heterogeneous clients [8]. However, an artistic prototype can itself reveal the styles or media contained in a local collection. Protecting only model parameters while transmitting prototypes, class supports, clipping statistics, or sparse coordinate patterns without protection leaves an incomplete privacy boundary. Conversely, perturbing every transmitted quantity with a fixed noise schedule can cause unacceptable utility loss, particularly for high-dimensional visual encoders.

Communication creates a second difficulty. Visual IoT devices commonly operate through constrained or intermittent uplinks, while modern image encoders contain millions of parameters. Sparsified model perturbation can reduce both message size and the dimensional effect of privacy noise [9]. Independent client-side sparsification, however, can produce incompatible coordinate supports and disclose which parameters are locally important. This complicates secure aggregation, whose purpose is to reveal only an aggregate rather than individual client messages [10]. A practical method must therefore coordinate sparsity without using current unprotected client updates, preserve enough descent information for convergence, and account for the privacy cost of every released statistic.

This paper proposes FedArtSense, a privacy-preserving federated learning framework for artistic image classification in visual IoT sensor networks. FedArtSense first introduces dual-space prototype guidance. A feature-space margin aligns local image representations with protected global class prototypes while preserving inter-class separation, and a decision-space consistency loss aligns the classification head with prototype-induced semantic probabilities. A locally calculated representation discrepancy determines the alignment strength for each client, allowing strongly shifted sensors to receive greater guidance without over-regularizing clients that are already consistent with the global model.

FedArtSense next provides a unified client-level privacy mechanism for model updates, prototype numerators, class-support values, and clipping feedback. Update and prototype contributions are separately clipped, perturbed through distributed Gaussian noise, and released only through secure aggregation. Private quantile feedback adjusts the clipping thresholds, while a progress-aware scheduler allocates more precise updates to later refinement rounds. Privacy loss is accumulated through Rényi differential privacy, following the composition principle in [11]. The design extends adaptive clipping [12] and time-adaptive privacy spending [13] to a joint model-and-prototype transcript.

To reduce uplink cost, the server constructs a shared sparse support from previously released protected aggregates. Each coordinate receives an importance score based on persistent update magnitude, directional consistency, and staleness. Most positions exploit coordinates with strong historical contributions, while a smaller exploration subset prevents permanent exclusion. Since the mask for round *t* depends only on protected information released before that round, it does not expose the structure of a current private update. All participating clients transmit the same ordered support, making the sparse messages directly compatible with secure aggregation. The compression ratio is adapted using protected optimization progress and the available communication budget.

The contributions of this work are fourfold. First, it formulates distributed artistic style and medium classification as a client-level private learning problem for visual IoT sensors, explicitly considering label skew and persistent acquisition-domain shift. Second, it develops heterogeneity-aware dual-space prototype learning in which semantic references, their supports, and model updates are protected within one privacy budget. Third, it introduces privacy-safe importance-aware sparsification that coordinates client supports without accessing current unprotected updates. The analysis establishes transcript-level (ϵ,δ)-differential privacy and characterizes convergence in terms of stochastic variation, client heterogeneity, clipping bias, prototype drift, retained projection energy, and privacy noise. Fourth, it evaluates the complete framework on WikiArt [14], ArtBench-10 [15], and BAM [4] under non-IID visual IoT partitions, sensor-domain shifts, bandwidth heterogeneity, and client dropout.

At the default client-level privacy target $\left(\epsilon ,\delta \right)=\left(6,10^{-5}\right)$, FedArtSense obtains accuracies of 64.2%, 81.2%, and 75.3% on WikiArt, ArtBench-10, and BAM, respectively. Our results exceed Fed-SMP by 4.8, 4.4, and 4.1 percentage points. On WikiArt, FedArtSense reduces cumulative uplink traffic from 87.1 GiB for dense private averaging to 11.5 GiB and reaches 95% of its final validation accuracy in 121 rounds. Our results alse further show a client-membership attack area under the curve of 0.508, close to random guessing. The ablation, privacy-utility, sensor-shift, and edge-device results indicate that the improvement arises from the interaction of protected prototype alignment, adaptive privacy control, and coordinated sparse aggregation rather than from relaxing the stated privacy requirement.

## 2. Related Work

### 2.1. Federated Learning Under Statistical and System Heterogeneity

Federated learning enables multiple clients to optimize a shared model without transferring their raw observations to a central repository. FedAvg established the standard iterative pattern of local optimization followed by weighted server aggregation [1]. Its performance can deteriorate when client objectives differ substantially, as occurs when visual IoT sensors observe different artistic styles, media, illumination conditions, and institutional collections. FedProx limits local deviation through a proximal regularizer [5], whereas SCAFFOLD estimates control variates to correct biased client updates [6]. MOON addresses representation inconsistency using model-level contrastive learning [7]. These methods improve general non-IID optimization but do not explicitly construct privacy-protected semantic references for distributed artistic classes.

Prototype-based learning offers a compact alternative to exchanging sample-level representations. FedProto communicates class prototypes to accommodate heterogeneous client models and distributions [8]. More recent studies have expanded knowledge transfer under data and model heterogeneity. FedKTL uses a server-side pretrained generator to produce prototypical image-vector pairs while reducing client upload requirements [16]. FedType introduces proxy models and uncertainty-based asymmetric reciprocity for architecture-heterogeneous clients [17]. Recurrent early-exit models adapt inference and training depth to heterogeneous client resources [18], while FedLMT employs low-rank model training to reduce computation and communication on constrained devices [19]. These approaches primarily address architecture or hardware differences and generally assume that transmitted knowledge summaries do not require a joint client-level privacy guarantee.

Recent federated representation methods also exploit pretrained models and adaptive aggregation. FedPHA learns heterogeneous client prompts for vision–language models [20]. Theoretical analysis has shown that increasing network width can mitigate part of the optimization error induced by heterogeneous data [21]. FedAWARE instead studies client-consensus dynamics and adjusts aggregation weights to improve convergence under heterogeneity [22]. These developments demonstrate that representation sharing and adaptive optimization are complementary. FedArtSense differs by targeting images collected by visual IoT sensors and by combining client-specific dual-space prototype alignment with privacy-protected prototype aggregation. Its feature-space margin preserves artistic category separation, its decision-space loss aligns classifier semantics, and its discrepancy-dependent coefficient avoids imposing the same regularization strength on clients with different levels of drift.

VFL+ treats the feature views held by other parties as privileged training information so that each participant can perform autonomous inference after vertically federated training [23]. This low-coupling objective addresses dependence among parties that share sample identities but hold disjoint features. FedArtSense instead assumes horizontal federation: each institution holds complete images and labels for disjoint samples. Its per-round upload contains $O(m_{t}+C\left(d_{z}+1\right))$ protected values after local optimization, whereas vertical methods require sample-aligned cross-party information during training. Resistance to client drift is supplied by discrepancy-scaled feature- and decision-space alignment rather than by privileged feature views. The two mechanisms are complementary in motivation but not directly comparable as numerical baselines because their data partitions, messages, and inference requirements differ.

### 2.2. Differential Privacy and Communication-Efficient Federation

Keeping images on local sensors does not by itself prevent model updates from revealing information about a participating collection. Differential privacy provides a formal mechanism-level guarantee against such inference [24]. Rényi differential privacy supports tighter accounting for repeated Gaussian mechanisms and subsampled training rounds [11]. Client-level private federated learning protects the presence or absence of a complete client dataset rather than a single record [2], but clipping and Gaussian perturbation can substantially reduce utility when updates are high-dimensional or heterogeneous.

Adaptive clipping estimates an update-norm quantile with a small additional privacy expenditure [12]. Time-adaptive privacy spending further shows that allocating privacy non-uniformly across training rounds can improve late-stage learning [13]. FedCEO recovers part of the semantic information disrupted by privacy noise through low-rank proximal processing of stacked client models [25]. Private heterogeneous federated optimization has also been studied without a trusted server, with error-optimal results established for convex inter-silo objectives [26]. These methods motivate adaptive privacy control, but they do not jointly calibrate private model updates, class-support values, and artistic prototypes within one transcript-level budget [27,28].

Secure aggregation prevents an honest-but-curious coordinator from inspecting individual client messages [10]. It complements differential privacy because secure aggregation hides intermediate contributions, whereas differential privacy protects released aggregates and final models. Fed-SMP combines client-level differential privacy with sparsified model perturbation to reduce both communication and perturbation dimension [9]. Exact-error compression provides another connection between distributed quantization and privacy noise [29]. Lossless gradient sparsification maps updates into a more compressible alternative space [30], while recent low-rank decomposition methods reduce communication through structured factorization [31]. FedSMU represents model changes through symbolic updates to improve communication efficiency and generalization [32]. FedSC privately exchanges representation correlation matrices for federated self-supervised learning [33].

Transmission-oriented protection addresses a different boundary. Hyperchaotic memristive encryption with DNA encoding can protect images while they are exchanged, but an authorized endpoint still receives decryptable pixels and assumes responsibility for centralized storage and analysis [34]. Reversible adversarial protection such as DP-TRAE can obscure a raw image from unauthorized models while permitting key-based recovery [35]. These mechanisms are practical when an institution accepts centralized, authorized access but wants confidentiality in transit or storage. Federated learning instead avoids raw-image transfer and produces a shared classifier; secure aggregation hides individual messages and differential privacy limits the influence of one client on the released transcript. Encryption or reversible perturbation can be layered before local storage, but they do not replace transcript-level privacy accounting.

A persistent difficulty is that independently selected sparse supports can expose client-specific structure or become incompatible with fixed-vector secure aggregation. FedArtSense constructs one shared importance mask from previously released protected aggregates, so current-round coordinate selection does not depend on unprotected client data. Temporal magnitude, directional consistency, and staleness jointly determine the mask, while an exploration subset prevents permanent coordinate exclusion. Unlike approaches that protect only model updates, the proposed privacy accountant composes the model mechanism, prototype mechanism, and private clipping statistics. This design provides a single client-level guarantee for the complete released transcript.

### 2.3. Computational Analysis of Artistic Images

Computational art analysis differs from ordinary photographic recognition because artistic images can depart from natural geometry, texture, color, and object appearance. Early large-scale work showed that learned visual features can identify photographic and artistic styles across diverse sources [3]. Residual networks were subsequently applied to artistic-style recognition on large painting collections [36]. The WikiArt task organization used by ArtGAN provides artist, genre, and style classification metadata for historical artworks [14]. BAM extends artistic analysis to contemporary creative media and supplies content, emotion, and medium attributes [4]. ArtBench-10 provides a balanced and standardized benchmark spanning ten artistic styles [15].

Recent research has broadened artistic image analysis beyond style classification. LAPIS introduces artwork images, aesthetic ratings, image attributes, and annotator attributes for personalized aesthetic assessment [37]. NADA uses vision–language and diffusion-model knowledge to detect objects in paintings with weak or zero bounding-box supervision [38]. These studies highlight the growing diversity of artwork-analysis tasks, but their conventional settings assume that the images can be collected and processed centrally.

Visual IoT deployments introduce a different constraint: cameras and institutional gateways collect artworks at distributed physical locations, and the resulting images may expose collection contents or exhibition context. Existing artistic image methods do not provide client-level privacy for distributed sensor collections, while general private federated methods do not model the unusually strong style and medium shifts found in art. FedArtSense connects these areas by treating artistic images as private sensor observations, aligning heterogeneous local representations through protected prototypes, and reducing uplink traffic through a secure-aggregation-compatible shared sparse support.

## 3. Preliminaries

### 3.1. Visual IoT Sensor Network and Learning Task

We consider a visual IoT network composed of *K* distributed clients, denoted by K={1,…,K}. The evaluated client is an institution-controlled edge gateway or local server connected to fixed RGB documentation or surveillance cameras; visitor-owned devices, hyperspectral conservation scanners, and direct training on a battery-powered camera are outside scope. Client *k* stores an artistic image dataset $D_{k}={\left\{\left(x_{k,i},y_{k,i}\right)\right\}}_{i=1}^{n_{k}}$, where $x_{k,i}\in R^{H\times W\times 3}$ is an RGB image, $y_{k,i}\in Y=\left\{1,\ldots ,C\right\}$ is a style or medium label, and $n_{k}=\left|D_{k}\right|$. Source frames may be captured at 1920×1080 pixels or higher, but local preprocessing produces a 224×224 model input. Frames are acquired event-wise or at intervals of 5–30 s and buffered locally; federated rounds are periodic gateway jobs rather than frame-synchronous updates. Camera-to-gateway connectivity is local Ethernet or Wi-Fi, while the modeled gateway-to-server uplinks are 0.8, 3, and 8 Mbit/s. The total sample size is $n=\sum_{k=1}^{K}n_{k}$, and the statistical weight of client *k* is $p_{k}=n_{k}/n$.

Raw images remain within the administrative boundary of the client that collected them. Only privacy-protected learning information can be exchanged with the coordinating edge server. This restriction is important because an artistic image may reveal private collection contents, artwork ownership, exhibition arrangements, acquisition activities, or contextual information captured around the artwork. The coordinating server is assumed to be honest in executing the prescribed protocol but curious about the information contained in client messages. External observers may also inspect the released global models. The communication channels are authenticated, while secure aggregation is used to prevent the server from observing an individual client’s unprotected contribution.

The local data are generally non-independent and non-identically distributed. Different sensors may observe distinct artistic movements, creators, exhibition themes, illumination conditions, viewpoints, and imaging devices. Let $C_{k}\subseteq Y$ denote the set of classes observed by client *k*, and let $n_{k,c}$ be the number of its images belonging to class *c*. The local class proportion is $q_{k,c}=n_{k,c}/n_{k}$, whereas the global class proportion is $q_{c}=\sum_{k=1}^{K}p_{k}q_{k,c}$. Therefore, both label-distribution skew and appearance-domain shift can occur simultaneously. We write the local data distribution as $P_{k}\left(x,y\right)$ and do not assume that $P_{k}=P_{j}$ for two distinct clients *k* and *j*. The model is parameterized by θ=(ω,ϕ), where $g_{\omega }$ is an image encoder and $h_{\phi }$ is a classification head. For an input image x, the prediction is $f_{\theta }\left(x\right)=h_{\phi }\left(g_{\omega }\left(x\right)\right)$. The encoder output is normalized as $z=g_{\omega }\left(x\right)/{∥g_{\omega }\left(x\right)∥}_{2}$, which makes representations produced by different clients comparable. The evaluated task is exclusively *C*-class artistic image classification.

### 3.2. Federated Optimization and Prototype Representation

Let $\ell (f_{\theta }\left(x\right),y)$ denote the supervised cross-entropy loss. The empirical risk of client *k* and the global federated objective are

(1) $$F_{k}\left(\theta \right)=\frac{1}{n_{k}}\sum_{i=1}^{n_{k}}\ell \;\left(f_{\theta }\left(x_{k,i}\right),y_{k,i}\right),\;\theta^{*}=\arg\min_{\theta }F\left(\theta \right),\;F\left(\theta \right)=\sum_{k=1}^{K}p_{k}F_{k}\left(\theta \right).$$

Training proceeds for *T* communication rounds. At the beginning of round *t*, the server holds the global parameters $\theta^{t}$ and selects a participating client set $S_{t}\subseteq K$. Each selected client initializes its local model with $\theta^{t}$ and performs *E* local epochs using learning rate η. The resulting local parameters are denoted by $\theta_{k}^{t,E}$, and the corresponding model update is $\Delta_{k}^{t}=\theta_{k}^{t,E}-\theta^{t}$. For selected-client normalization, the aggregation weight is $p_{k,t}=n_{k}/\sum_{j\in S_{t}}n_{j}$. Conventional weighted aggregation can be expressed as

(2) $$\theta^{t+1}=\theta^{t}+\sum_{k\in S_{t}}p_{k,t}\Delta_{k}^{t}.$$

Under severe statistical heterogeneity, however, minimizing each $F_{k}$ independently can move local representations toward incompatible optima. This phenomenon is especially pronounced when a sensor observes only a small subset of artistic styles. Consequently, the proposed method augments the task loss with prototype-guided representation alignment while retaining Equation (1) as the underlying learning problem.

For each observed class $c\in C_{k}$, client *k* summarizes its local representation space using the class prototype

(3) $$\mu_{k,c}^{t}=\frac{\sum_{i=1}^{n_{k}}I\left(y_{k,i}=c\right)\;z_{k,i}^{t}}{n_{k,c}},\;z_{k,i}^{t}=\frac{g_{\omega_{k}^{t,E}}\left(x_{k,i}\right)}{{∥g_{\omega_{k}^{t,E}}\left(x_{k,i}\right)∥}_{2}}.$$

Here, I(·) is the indicator function. A prototype is not constructed when $n_{k,c}=0$. Let $S_{t,c}=\left\{k\in S_{t}:c\in C_{k}\right\}$ be the clients that observe class *c* in round *t*. Before privacy protection, their weighted reference prototype would be

(4) $${\bar{\mu }}_{c}^{t}=\frac{\sum_{k\in S_{t,c}}n_{k,c}\mu_{k,c}^{t}}{\sum_{k\in S_{t,c}}n_{k,c}}.$$

These class-level summaries contain substantially fewer dimensions than the complete model, but they can still reveal properties of a local collection. They must therefore be incorporated into the same privacy-protected communication mechanism as the model updates. In the remainder of the paper, ${\hat{\Delta }}_{k}^{t}$ and ${\hat{\mu }}_{k,c}^{t}$ denote a protected update and a protected prototype, respectively.

### 3.3. Privacy and Communication Criteria

The privacy target is defined at the client level. Two federated datasets $D=\{D_{1},\ldots ,D_{K}\}$ and $D^{\prime }$ are adjacent, written as $D≃D^{\prime }$, if one can be obtained from the other by adding or removing the complete dataset of one client. This definition protects whether an institution or sensor collection participated in training, including all artistic images associated with that participant. A randomized federated mechanism M satisfies (ϵ,δ)-client-level differential privacy if, for every adjacent pair $D≃D^{\prime }$ and every measurable output set O,

(5) $$\mathrm{Pr}\left[M\left(D\right)\in O\right]\leq e^{\epsilon }\mathrm{Pr}\left[M\left(D^{\prime }\right)\in O\right]+\delta ,$$

where ϵ controls the privacy loss and δ bounds the probability of exceptional disclosure. A smaller ϵ indicates stronger privacy for a fixed δ.

To bound the contribution of a selected client, an update vector v is projected onto an $\ell_{2}$ ball using $\mathrm{Clip}\left(v,R\right)=v\min\{1,R/∥v{∥}_{2}\}$, where R>0 is the clipping threshold. A Gaussian mechanism releases a clipped aggregate perturbed by zero-mean noise whose standard deviation is proportional to *R*. FedArtSense uses round-dependent thresholds $R_{t}$ and noise multipliers $\sigma_{t}$ so that privacy resources follow training sensitivity rather than being assigned uniformly.

Privacy accumulation is tracked through Rényi differential privacy. At Rényi order λ>1, let $\rho_{t}\left(\lambda \right)$ denote the privacy cost of round *t*. Adaptive composition gives $\rho_{1:T}\left(\lambda \right)=\sum_{t=1}^{T}\rho_{t}\left(\lambda \right)$. For any chosen δ∈(0,1), the corresponding privacy bound is

(6) $$\epsilon =\min_{\lambda >1}\left\{\rho_{1:T}\left(\lambda \right)+\frac{\log(1/\delta )}{\lambda -1}\right\}.$$

Client subsampling is included in $\rho_{t}\left(\lambda \right)$, with participation rate $r_{t}=\left|S_{t}\right|/K$. The complete released transcript, including the sequence of global models and global class prototypes, is accounted for within the same total privacy budget (ϵ,δ).

Communication efficiency is evaluated by the number of transmitted bits rather than only by the number of rounds. Let d=|θ| be the model dimension, $m_{k}^{t}$ be the number of update coordinates transmitted by client *k* in round *t*, and *b* be the bit width of each retained value. Encoding an index requires $\left\lceil \log_{2}d\right\rceil$ bits. If $B_{\mathrm{proto}}$ denotes the total cost of transmitting protected prototypes and their class identifiers, the overall client-to-server communication cost is

(7) $$B_{\mathrm{total}}=\sum_{t=0}^{T-1}\sum_{k\in S_{t}}m_{k}^{t}\left(b+\left\lceil \log_{2}d\right\rceil \right)+B_{\mathrm{proto}}.$$

The compression ratio is $\gamma_{k}^{t}=m_{k}^{t}/d$. The methodology uses importance-aware sparsification to reduce $\gamma_{k}^{t}$ while preserving coordinates that contribute most strongly to global optimization. Accordingly, the learning problem is evaluated through predictive performance, robustness to heterogeneous client distributions, cumulative client-level privacy loss, and total communication cost.

## 4. Methodology

### 4.1. FedArtSense Overview and Prototype-Guided Local Learning

**Figure 1:**
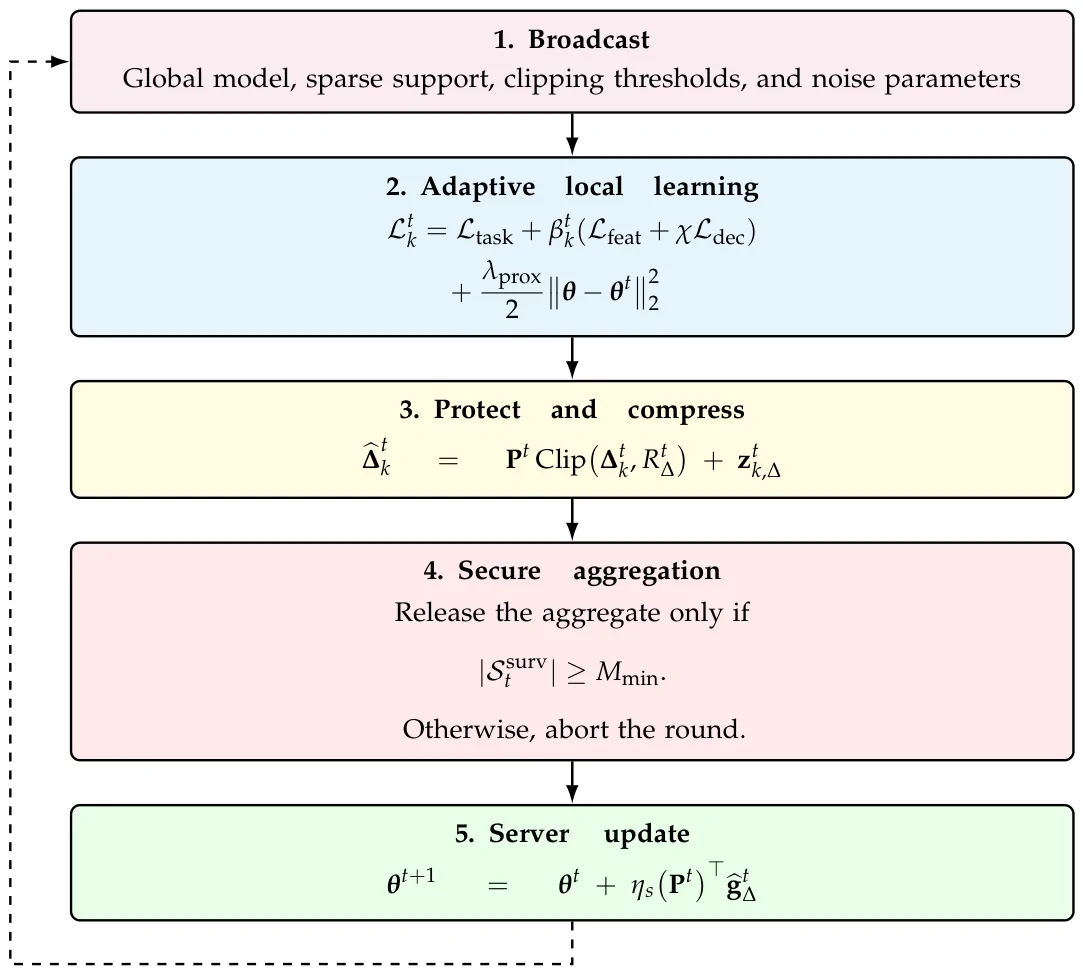
FedArtSense privacy-preserving federated learning workflow.

**Figure 2:**
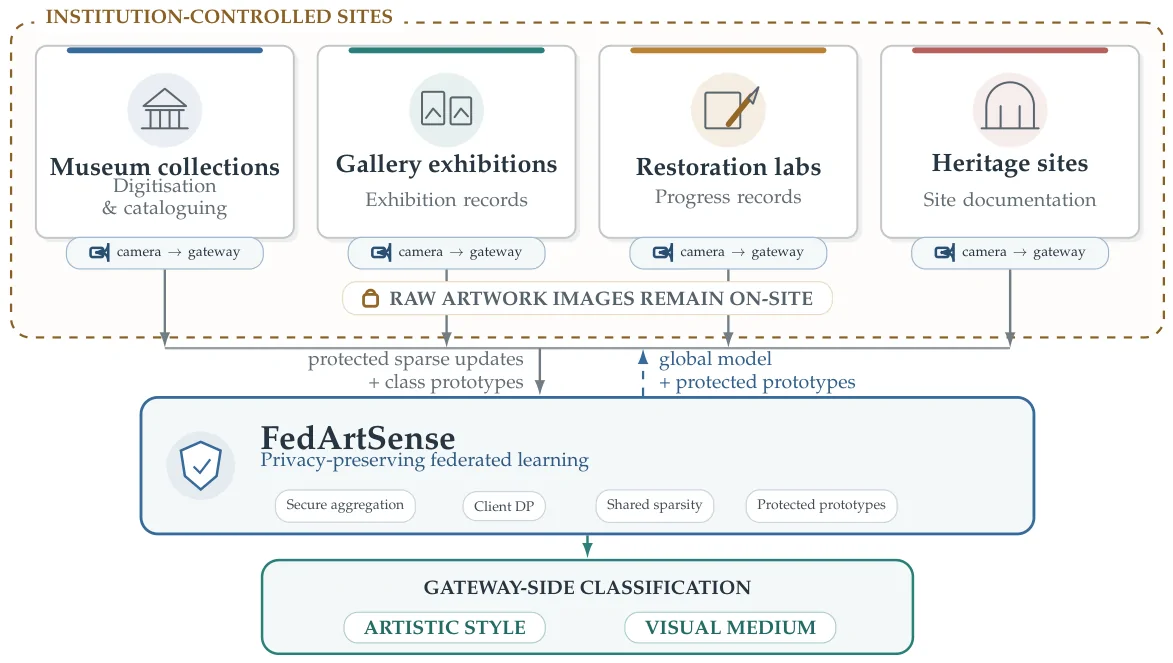
Application scenarios for FedArtSense. Fixed RGB cameras in museums, galleries, restoration laboratories, and heritage or public exhibition sites buffer artwork images at institutional gateways. Raw images remain local; only privacy-protected sparse model updates and class prototypes are securely aggregated. The updated model supports local artistic style and visual-medium classification.

FedArtSense jointly addresses statistical heterogeneity, privacy leakage, and communication constraints in visual IoT sensor networks. Figure 1 shows a general pipeline of our proposal. Museums, galleries, and heritage sites retain camera frames and labels on institution-controlled gateways while exchanging only protected sparse model and prototype messages. The round-level pipeline combines discrepancy-adaptive dual-space learning, joint clipping and distributed noise, survivor-gated secure aggregation, and privacy-accounted server adaptation. The displayed equations summarize the local objective, protected client message, and global model update. Figure 1 and Figure 2 show the general pipeline and application scenario of our proposal.

Each communication round contains four coupled operations: prototype-guided local optimization, adaptive privacy protection, importance-aware update sparsification, and secure server aggregation. At the beginning of round *t*, the server broadcasts the current global model $\theta^{t}$ and a protected global prototype set ${\hat{P}}^{t}=\left\{{\hat{\bar{\mu }}}_{c}^{t}:c\in G_{t}\right\}$, where $G_{t}\subseteq Y$ contains the classes for which a global reference prototype is available. At t=0, ${\hat{P}}^{0}$ is empty, and clients perform one warm-up round using only the supervised task objective. The principal difficulty of artistic image learning in distributed sensor networks is that clients can have substantially different visual and label distributions. Directly minimizing local empirical risk can consequently produce incompatible representation spaces. FedArtSense addresses this problem by aligning each local representation with protected class-level knowledge rather than transferring raw images or sample-level features.

Before local optimization, client *k* uses the received encoder $g_{\omega^{t}}$ to compute an initial prototype for every locally observed class. For $c\in C_{k}$, this prototype is

(8) $$\begin{array}{cccc} \nu_{k,c}^{t}\; & =\frac{\sum_{i=1}^{n_{k}}I\left(y_{k,i}=c\right)\;z_{k,i}^{t,0}}{{∥\sum_{i=1}^{n_{k}}I\left(y_{k,i}=c\right)\;z_{k,i}^{t,0}∥}_{2}+\upsilon },\; & z_{k,i}^{t,0}\; & =\frac{g_{\omega^{t}}\left(x_{k,i}\right)}{{∥g_{\omega^{t}}\left(x_{k,i}\right)∥}_{2}+\upsilon }. \end{array}$$

The initial prototypes are retained locally and are not transmitted. Let $A_{k}^{t}=C_{k}\cap G_{t}$ denote the set of classes shared by the local dataset and the protected global prototype set. FedArtSense measures the client-specific representation discrepancy as

(9) $$d_{k}^{t}=\left\{\begin{array}{cc} \frac{1}{|A_{k}^{t}|}\sum_{c\in A_{k}^{t}}\frac{1-{\left(\nu_{k,c}^{t}\right)}^{⊤}{\hat{\bar{\mu }}}_{c}^{t}}{2},\; & |A_{k}^{t}|>0,\\ 0, & |A_{k}^{t}|=0. \end{array}\right.$$

Because both vectors are $\ell_{2}$-normalized, $d_{k}^{t}\in \left[0,1\right]$. A value close to zero indicates that local and global class representations are already consistent, whereas a larger value indicates stronger client drift. The discrepancy remains on the client and therefore creates no additional communication or privacy cost.

A fixed prototype regularization strength may over-constrain clients whose distributions are already close to the global model and under-correct clients with severe domain shift. FedArtSense instead introduces the heterogeneity-aware coefficient

(10) $$\beta_{k}^{t}=\beta_{\max}\frac{d_{k}^{t}}{d_{k}^{t}+\xi },$$

where $\beta_{\max}>0$ is the maximum alignment strength and ξ>0 controls its sensitivity. During the warm-up round or when $A_{k}^{t}$ is empty, $\beta_{k}^{t}=0$.

For a local mini-batch $B_{k,e}^{t}$ at local step *e*, the supervised classification loss is

(11) $$L_{\mathrm{task},k}^{t}=-\frac{1}{|B_{k,e}^{t}|}\sum_{\left(x,y\right)\in B_{k,e}^{t}}\log p_{\theta }\left(y|x\right),$$

where $p_{\theta }\left(c|x\right)$ is the softmax output of $f_{\theta }$. To align the encoder without collapsing visually distinct artistic categories, FedArtSense employs a margin-based prototype loss. Let $z=g_{\omega }\left(x\right)/(∥g_{\omega }\left(x\right){∥}_{2}+\upsilon )$, let ${\left[a\right]}_{+}=\max\left(0,a\right)$, and let m>0 be a cosine margin. For a sample whose label *y* belongs to $G_{t}$, its feature-space alignment loss is

(12) $$\begin{array}{cc} L_{\mathrm{feat}}\left(x,y\right)\; & =1-z^{⊤}{\hat{\bar{\mu }}}_{y}^{t}\\ \; & \;+\frac{1}{|G_{t}|-1}\sum_{\begin{array}{c} c\in G_{t}\\ c\neq y \end{array}}{\left[m+z^{⊤}{\hat{\bar{\mu }}}_{c}^{t}-z^{⊤}{\hat{\bar{\mu }}}_{y}^{t}\right]}_{+}. \end{array}$$

The first term attracts an image representation toward the protected prototype of its true class. The second requires the true-class similarity to exceed every available competing-class similarity by at least *m*. This construction is useful for artistic categories with similar color palettes or compositional structures, for which simple attraction may not produce sufficiently discriminative features.

Feature alignment alone does not guarantee that the local classification head interprets shared representation geometry consistently. Given temperature τ>0, the protected prototypes define the reference posterior:

(13) $$r_{t}\left(c|z\right)=\frac{\exp\;\left(z^{⊤}{\hat{\bar{\mu }}}_{c}^{t}/\tau \right)}{\sum_{j\in G_{t}}\exp\;\left(z^{⊤}{\hat{\bar{\mu }}}_{j}^{t}/\tau \right)},\;c\in G_{t}.$$

The local model posterior is restricted and renormalized over the same class set as

(14) $${\tilde{p}}_{\theta }\left(c|x\right)=\frac{p_{\theta }\left(c|x\right)}{\sum_{j\in G_{t}}p_{\theta }\left(j|x\right)},\;c\in G_{t}.$$

The decision-space consistency loss is

(15) $$L_{\mathrm{dec}}\left(x\right)=\sum_{c\in G_{t}}\mathrm{sg}\left[r_{t}\left(c|z\right)\right]\log\frac{\mathrm{sg}[r_{t}\left(c|z\right)]}{{\tilde{p}}_{\theta }\left(c|x\right)},$$

where sg[·] is the stop-gradient operator. It prevents the reference posterior from moving toward an incorrect local prediction during the same optimization step. Equations (12) and (15) form a dual-space alignment mechanism: the former preserves class geometry in the encoder space, while the latter transfers that geometry to the classifier output.

For samples whose labels do not yet have global prototypes, both alignment terms are set to zero. Averaging the valid terms within $B_{k,e}^{t}$ gives $L_{\mathrm{feat},k}^{t}$ and $L_{\mathrm{dec},k}^{t}$. The complete local objective is

(16) $$L_{k}^{t}\left(\theta \right)=L_{\mathrm{task},k}^{t}+\beta_{k}^{t}\left(L_{\mathrm{feat},k}^{t}+\chi L_{\mathrm{dec},k}^{t}\right)+\frac{\lambda_{\mathrm{prox}}}{2}{∥\theta -\theta^{t}∥}_{2}^{2},$$

where χ≥0 balances feature-space and decision-space alignment, and $\lambda_{\mathrm{prox}}\geq 0$ limits excessive local deviation. Starting from $\theta_{k}^{t,0}=\theta^{t}$, the client performs

(17) $$\theta_{k}^{t,e+1}=\theta_{k}^{t,e}-\eta \nabla_{\theta }L_{k}^{t}\left(\theta_{k}^{t,e}\right),\;e=0,\ldots ,E_{k}^{t}-1.$$

The number of local steps $E_{k}^{t}$ may vary with the available computing capacity of client *k*. After optimization, the client obtains $\Delta_{k}^{t}=\theta_{k}^{t,E_{k}^{t}}-\theta^{t}$ and constructs final local prototypes using Equation (3). These outputs are processed by the privacy and communication modules before leaving the client.

### 4.2. Adaptive Client-Level Privacy Protection

The local model update $\Delta_{k}^{t}$ and class prototypes ${\left\{\mu_{k,c}^{t}\right\}}_{c\in C_{k}}$ contain information about the artistic images observed by client *k*. FedArtSense protects both types of information under the client-level adjacency relation defined in Section 3.3. The mechanism combines adaptive contribution clipping, distributed Gaussian perturbation, private threshold calibration, and Rényi privacy accounting. All adaptation variables used in round *t* are derived from previously released private statistics; consequently, their use does not introduce an unaccounted dependence on the current private dataset.

For model aggregation, the contribution of client *k* is weighted by $p_{k,t}$ and bounded using the round-dependent threshold $R_{\Delta }^{t}$:

(18) $$u_{k,\Delta }^{t}=\mathrm{Clip}\;\left(p_{k,t}\Delta_{k}^{t},R_{\Delta }^{t}\right)=p_{k,t}\Delta_{k}^{t}\min\left\{1,\frac{R_{\Delta }^{t}}{∥p_{k,t}\Delta_{k}^{t}{∥}_{2}}\right\}.$$

It follows that ${∥u_{k,\Delta }^{t}∥}_{2}\leq R_{\Delta }^{t}$. A public coordinate projection $P^{t}\in {\left\{0,1\right\}}^{m_{t}\times d}$ retains $m_{t}$ model coordinates selected from information released before round *t*. Its construction is presented in Section 4.3. Because $P^{t}$ is independent of the current round’s private data and satisfies $∥P^{t}{v∥}_{2}\leq {∥v∥}_{2}$, projection does not increase sensitivity.

The prototype payload preserves the numerator and denominator required to construct a weighted global class prototype. Client *k* defines $q_{k,c}=n_{k,c}/n_{k}$ for $c\in C_{k}$ and sets $q_{k,c}=0$ otherwise. Its weighted payload is

(19) $$v_{k}^{t}=\mathrm{vec}\left({\left\{p_{k,t}q_{k,c}\mu_{k,c}^{t},\;p_{k,t}q_{k,c}\right\}}_{c=1}^{C}\right),$$

where vec(·) concatenates the class-wise prototype numerators and supports. Support values are protected because an unprotected class count could disclose which artistic styles or media are present in a collection. The bounded prototype contribution is

(20) $$u_{k,P}^{t}=\mathrm{Clip}\left(v_{k}^{t},R_{P}^{t}\right),$$

where $R_{P}^{t}>0$ is the prototype clipping threshold.

Thresholds that are unnecessarily large increase noise, whereas thresholds that are too small introduce clipping bias. FedArtSense calibrates $R_{\Delta }^{t}$ and $R_{P}^{t}$ toward a target unclipped fraction $q^{*}\in \left(0,1\right)$. Each participating client computes

(21) $$\begin{array}{cc} a_{k,\Delta }^{t} & =I\;\left(∥p_{k,t}\Delta_{k}^{t}{∥}_{2}\leq R_{\Delta }^{t}\right),\\ a_{k,P}^{t} & =I\;\left(∥v_{k}^{t}{∥}_{2}\leq R_{P}^{t}\right). \end{array}$$

These binary values are securely aggregated and perturbed before release. Let $M_{t}=\left|S_{t}\right|$ and let $\zeta_{\Delta }^{t}$ and $\zeta_{P}^{t}$ be Gaussian random variables generated through distributed noise shares. The released unclipped-fraction estimates are

(22) $$\begin{array}{cc} {\tilde{q}}_{\Delta }^{t} & ={\mathrm{clip}}_{\left[0,1\right]}\;\left(\frac{\sum_{k\in S_{t}}a_{k,\Delta }^{t}+\zeta_{\Delta }^{t}}{M_{t}}\right),\\ {\tilde{q}}_{P}^{t} & ={\mathrm{clip}}_{\left[0,1\right]}\;\left(\frac{\sum_{k\in S_{t}}a_{k,P}^{t}+\zeta_{P}^{t}}{M_{t}}\right). \end{array}$$

With adaptation rate $\kappa_{R}>0$, the thresholds used in the next round are updated in the logarithmic domain:

(23) $$\begin{array}{cc} R_{\Delta }^{t+1} & ={\mathrm{clip}}_{\left[R_{\Delta }^{\min},R_{\Delta }^{\max}\right]}\;\left[R_{\Delta }^{t}\exp\;\left(\kappa_{R}\left(q^{*}-{\tilde{q}}_{\Delta }^{t}\right)\right)\right],\\ R_{P}^{t+1} & ={\mathrm{clip}}_{\left[R_{P}^{\min},R_{P}^{\max}\right]}\;\left[R_{P}^{t}\exp\;\left(\kappa_{R}\left(q^{*}-{\tilde{q}}_{P}^{t}\right)\right)\right]. \end{array}$$

Thus, a low observed unclipped fraction increases the threshold, while a high fraction tightens the sensitivity bound.

FedArtSense adopts distributed Gaussian perturbation so that the server observes only noisy sums through secure aggregation. Let $M_{\min}$ be the minimum number of clients required to complete a round. Each participating client generates independent noise shares

(24) $$\begin{array}{cc} z_{k,\Delta }^{t} & ∼N\;\left(0,\frac{{\left(\sigma_{\Delta }^{t}R_{\Delta }^{t}\right)}^{2}}{M_{\min}}I_{m_{t}}\right),\\ z_{k,P}^{t} & ∼N\;\left(0,\frac{{\left(\sigma_{P}^{t}R_{P}^{t}\right)}^{2}}{M_{\min}}I_{C(d_{z}+1)}\right), \end{array}$$

where $d_{z}$ is the feature dimension and $\sigma_{\Delta }^{t}$ and $\sigma_{P}^{t}$ are round-dependent noise multipliers. Protected contributions supplied to secure aggregation are

(25) $$\begin{array}{cc} {\hat{\Delta }}_{k}^{t} & =P^{t}u_{k,\Delta }^{t}+z_{k,\Delta }^{t},\\ {\hat{v}}_{k}^{t} & =u_{k,P}^{t}+z_{k,P}^{t}. \end{array}$$

If at least $M_{\min}$ clients complete the protocol, aggregate noise covariance is no smaller than ${\left(\sigma_{\Delta }^{t}R_{\Delta }^{t}\right)}^{2}I_{m_{t}}$ for the model and ${\left(\sigma_{P}^{t}R_{P}^{t}\right)}^{2}I_{C(d_{z}+1)}$ for the prototype payload. A round is aborted without releasing an aggregate when fewer than $M_{\min}$ noise shares are available.

A fixed noise multiplier assigns equal privacy expenditure to early unstable rounds and late refinement rounds. FedArtSense instead adjusts per-round expenditure using a convergence signal calculated from previously released private global models. For t≥1, the public progress score is

(26) $$g_{t}=\frac{∥\theta^{t}-\theta^{t-1}{∥}_{2}}{{∥\theta^{t-1}∥}_{2}+\upsilon },\;{\bar{g}}_{t}=\varrho {\bar{g}}_{t-1}+\left(1-\varrho \right)g_{t},$$

where υ>0 prevents division by zero and ϱ∈[0,1). Let $\rho_{\mathrm{rem}}^{t}\left(\lambda \right)$ be the remaining Rényi privacy budget at order λ. The target expenditure is

(27) $$\rho_{\mathrm{tar}}^{t}\left(\lambda \right)=\min\left\{\rho_{\max}\left(\lambda \right),\frac{\rho_{\mathrm{rem}}^{t}\left(\lambda \right)}{T-t}{\mathrm{clip}}_{\left[a_{\min},a_{\max}\right]}\;\left(\frac{{\bar{g}}_{t}+\upsilon }{g_{t}+\upsilon }\right)\right\}.$$

Smaller late-stage model movement therefore receives a larger Rényi allocation and a smaller noise multiplier, while the privacy filter prevents cumulative expenditure from exceeding the prescribed total budget.

The target cost is divided among the model update, prototype payload, and two clipping-statistic mechanisms using shares $\pi_{\Delta }$, $\pi_{P}$, and $\pi_{q}$, where $\pi_{\Delta }+\pi_{P}+2\pi_{q}=1$. Let $A_{\mathrm{SGM}}\left(\lambda ,r_{t},\sigma \right)$ denote the Rényi cost of a client-subsampled Gaussian mechanism. The multipliers are the smallest values satisfying

(28) $$\begin{array}{cc} A_{\mathrm{SGM}}\left(\lambda ,r_{t},\sigma_{\Delta }^{t}\right) & \leq \pi_{\Delta }\rho_{\mathrm{tar}}^{t}\left(\lambda \right),\\ A_{\mathrm{SGM}}\left(\lambda ,r_{t},\sigma_{P}^{t}\right) & \leq \pi_{P}\rho_{\mathrm{tar}}^{t}\left(\lambda \right),\\ A_{\mathrm{SGM}}\left(\lambda ,r_{t},\sigma_{q}^{t}\right) & \leq \pi_{q}\rho_{\mathrm{tar}}^{t}\left(\lambda \right). \end{array}$$

The multiplier $\sigma_{q}^{t}$ controls each noisy clipping indicator. In practice, these inequalities are solved over a fixed grid of Rényi orders. At order λ>1, the privacy cost of round *t* is bounded by

(29) $$\begin{array}{cc} \rho_{t}\left(\lambda \right) & =A_{\mathrm{SGM}}\left(\lambda ,r_{t},\sigma_{\Delta }^{t}\right)+A_{\mathrm{SGM}}\left(\lambda ,r_{t},\sigma_{P}^{t}\right)\\ \; & \;+2A_{\mathrm{SGM}}\left(\lambda ,r_{t},\sigma_{q}^{t}\right). \end{array}$$

Adaptive composition remains valid because the thresholds, projection matrices, and multipliers for round *t* depend only on previously released differentially private outputs. After *T* rounds, all released global models, protected prototypes, clipping statistics, and deterministic post-processing operations satisfy (ϵ,δ)-client-level differential privacy with

(30) $$\epsilon =\min_{\lambda >1}\left\{\sum_{t=0}^{T-1}\rho_{t}\left(\lambda \right)+\frac{\log(1/\delta )}{\lambda -1}\right\}.$$

This bound covers the complete training transcript rather than only the final global model.

The subsampled-Gaussian Rényi kernel follows the analytical bound of Wang et al. [39]. The public implementation will expose the per-round sampling rate and noise multipliers and include constant-schedule regression tests against the Opacus RDP accountant [40]. The shares $\left(\pi_{\Delta },\pi_{P},\pi_{q}\right)=\left(0.72,0.18,0.05\right)$ are a fixed heuristic rather than a dataset-specific optimum: 90% of each round’s Rényi allocation is assigned to the model and prototype vectors, and the remaining 10% is divided between the two clipping indicators.

Client-level differential privacy applies jointly to the temporal sequence of models, noisy class supports, and protected prototypes, including an adversary with auxiliary information. It limits the additional evidence attributable to one client’s participation, but it does not conceal population-level facts shared by many clients. A long prototype sequence can therefore reveal that a style or medium is common across the federation even when no individual collection is identifiable. The support threshold $s_{\min}$ and temporal smoothing suppress unstable low-support releases, but prototype-specific attribute inference is not separately evaluated; the attack AUC measures client membership from model statistics.

### 4.3. Importance-Aware Sparsification and Secure Aggregation

Transmitting a dense model update is expensive for visual IoT clients with limited uplink bandwidth. Independent top-*m* sparsification can reduce this cost, but its coordinate indices may reveal client-specific gradient structure and prevent efficient secure aggregation because clients transmit different supports. FedArtSense instead constructs a shared sparse projection from previously released private global updates. The projection is fixed before the current local datasets are accessed, so every participating client transmits the same coordinate positions and selection incurs no additional privacy loss.

Let ${\hat{g}}_{\Delta }^{t-1}\in R^{m_{t-1}}$ denote the protected sparse aggregate released in round t−1. The server lifts this vector to the original parameter space using

(31) $${\bar{\Delta }}^{t-1}={\left(P^{t-1}\right)}^{⊤}{\hat{g}}_{\Delta }^{t-1}\in R^{d}.$$

Coordinates not selected in round t−1 are zero after lifting. Because ${\hat{g}}_{\Delta }^{t-1}$ has already been protected, any score computed from ${\bar{\Delta }}^{t-1}$ is post-processing.

FedArtSense evaluates coordinate importance using persistent update magnitude and directional consistency. The server maintains an exponential moving average $a^{t}\in R^{d}$ of normalized magnitudes:

(32) $$a_{j}^{t}=\beta_{a}a_{j}^{t-1}+\left(1-\beta_{a}\right)\frac{|{\bar{\Delta }}_{j}^{t-1}|}{{∥{\bar{\Delta }}^{t-1}∥}_{1}+\upsilon },\;j=1,\ldots ,d,$$

where $\beta_{a}\in \left[0,1\right)$ controls temporal smoothing. A global update momentum is

(33) $$m_{j}^{t}=\beta_{m}m_{j}^{t-1}+\left(1-\beta_{m}\right){\bar{\Delta }}_{j}^{t-1},$$

where $\beta_{m}\in \left[0,1\right)$. For a coordinate selected in the preceding round, directional consistency is

(34) $$c_{j}^{t}=\frac{1}{2}\left[1+\mathrm{sign}\left({\bar{\Delta }}_{j}^{t-1}\right)\mathrm{sign}\left(m_{j}^{t-1}\right)\right].$$

Thus, $c_{j}^{t}=1$ when the most recent protected update agrees with its historical momentum, $c_{j}^{t}=0$ when their directions conflict, and $c_{j}^{t}=1/2$ when either value is zero. For an unselected coordinate, $c_{j}^{t}$ is retained from its most recent observation.

Pure magnitude selection can repeatedly choose a small parameter subset and permanently exclude coordinates that become useful later. The server therefore associates coordinate *j* with a staleness counter $h_{j}^{t}$. Let $M_{t-1}$ be the preceding support. The counter evolves as

(35) $$h_{j}^{t}=\left\{\begin{array}{cc} 0, & j\in M_{t-1},\\ \min(h_{j}^{t-1}+1,h_{\max}), & j\notin M_{t-1}, \end{array}\right.$$

where $h_{\max}$ limits the effect of long exclusion. The complete importance score is

(36) $$I_{j}^{t}=a_{j}^{t}\left(1+\kappa_{c}c_{j}^{t}\right)+\kappa_{h}\frac{h_{j}^{t}}{h_{\max}},$$

where $\kappa_{c}\geq 0$ rewards directionally consistent coordinates and $\kappa_{h}\geq 0$ promotes stale coordinates.

The number of retained coordinates is adapted to optimization progress and the available communication budget. Let $B_{\mathrm{round}}^{t}$ be the maximum uplink bits available in round *t*, let *b* be value precision, and let $B_{P}^{t}$ be protected-prototype transmission cost. A budget-feasible upper bound is

(37) $$m_{\mathrm{bud}}^{t}=\left\lfloor \frac{B_{\mathrm{round}}^{t}-B_{P}^{t}}{M_{t}\left(b+\left\lceil \log_{2}d\right\rceil \right)}\right\rfloor .$$

The desired compression ratio is derived from the public progress score:

(38) $$\gamma_{t}={\mathrm{clip}}_{\left[\gamma_{\min},\gamma_{\max}\right]}\;\left[\gamma_{0}{\left(\frac{g_{t}+\upsilon }{{\bar{g}}_{t}+\upsilon }\right)}^{\kappa_{\gamma }}\right],\;m_{t}=\min\left\{\left\lceil \gamma_{t}d\right\rceil ,m_{\mathrm{bud}}^{t}\right\}.$$

More coordinates are retained when the protected global model changes rapidly, while stronger compression is applied as optimization stabilizes. Setting $\gamma_{\min}=\gamma_{\max}$ yields a fixed-bandwidth evaluation.

A fraction $\gamma_{\exp}\in \left[0,1\right)$ of the support is reserved for exploration, with $m_{\exp}^{t}=\left\lceil \gamma_{\exp}m_{t}\right\rceil$. FedArtSense first chooses coordinates with the largest staleness values and then fills the remaining positions using importance:

(39) $$\begin{array}{cc} M_{t}^{\exp} & =\mathrm{Top}({\left\{h_{j}^{t}\right\}}_{j=1}^{d},m_{\exp}^{t}),\\ M_{t}^{\mathrm{imp}} & =\mathrm{Top}(\left\{I_{j}^{t}:j\notin M_{t}^{\exp}\right\},m_{t}-m_{\exp}^{t}),\\ M_{t} & =M_{t}^{\exp}\cup M_{t}^{\mathrm{imp}}. \end{array}$$

Here, Top(·,m) returns the indices of the *m* largest values, with ties resolved using a public round seed. Projection matrix $P^{t}$ contains one row for each index in $M_{t}$. Thus, $P^{t}v$ extracts selected coordinates and ${\left(P^{t}\right)}^{⊤}$ restores them to their original positions with zeros elsewhere.

The server broadcasts $M_{t}$ together with $\theta^{t}$, ${\hat{P}}^{t}$, $R_{\Delta }^{t}$, $R_{P}^{t}$, and the noise multipliers. Because all clients use the same ordered support, secure aggregation processes a fixed-length vector without observing client-specific coordinate indices. Equation (37) conservatively includes an index for every value so that comparisons remain valid for baselines with uncoordinated supports. An implementation that caches the shared support transmits only $m_{t}$ ordered values per client.

For protocol-level accounting, FedArtSense uses the four-phase, double-masking protocol of Bonawitz et al. [10]. Pairwise secrets use 32-byte Curve25519 public keys, authenticated share transport uses a 12-byte nonce and 16-byte tag, and the two 32-byte mask seeds are Shamir-shared with threshold $M_{\min}$. Protected values are encoded in a 32-bit fixed-point ring. Mutually cancelling pairwise masks and one self-mask are expanded from the exchanged seeds, so full mask vectors are not transmitted. Dropout-recovery shares remove masks associated with unavailable clients without exposing a surviving client’s message; fewer than $M_{\min}$ survivors causes the round to abort.

For $M_{t}$ selected clients, the bidirectional control-plane model is

(40) $$B_{\mathrm{ctrl}}\left(M_{t}\right)=64+64\left(M_{t}-1\right)+2\times 92\left(M_{t}-1\right)+32M_{t}\;\mathrm{bytes}/\mathrm{client}.$$

The terms respectively represent the key upload, peer-key broadcast, encrypted share upload/download, and worst-case unmasking packet. The uplink-only part is $B_{\mathrm{ctrl}}^{↑}\left(M_{t}\right)=64+92\left(M_{t}-1\right)+32M_{t}$. Three server-mediated control exchanges add approximately $3\;\mathrm{RTT}+8B_{\mathrm{ctrl}}/R$ seconds at link rate *R*. The masked model/prototype vector itself is already included in the payload accounting of Equation (7).

The protected sparse model aggregate is

(41) $${\hat{g}}_{\Delta }^{t}=\mathrm{SecAgg}\;\left({\left\{{\hat{\Delta }}_{k}^{t}\right\}}_{k\in S_{t}}\right)=\sum_{k\in S_{t}}{\hat{\Delta }}_{k}^{t}.$$

After lifting, the server updates the model using learning rate $\eta_{s}$:

(42) $$\theta^{t+1}=\theta^{t}+\eta_{s}{\left(P^{t}\right)}^{⊤}{\hat{g}}_{\Delta }^{t}.$$

When $\gamma_{t}=1$, clipping is inactive, noise is absent, and $\eta_{s}=1$, this reduces to Equation (2). Under compression, parameters outside $M_{t}$ retain their previous values until selected later.

The prototype vectors are aggregated independently:

(43) $${\hat{g}}_{P}^{t}=\mathrm{SecAgg}\;\left({\left\{{\hat{v}}_{k}^{t}\right\}}_{k\in S_{t}}\right)=\sum_{k\in S_{t}}{\hat{v}}_{k}^{t}.$$

The server decomposes ${\hat{g}}_{P}^{t}$ into noisy numerator ${\hat{a}}_{c}^{t}\in R^{d_{z}}$ and noisy support ${\hat{s}}_{c}^{t}$ for each class. A candidate protected prototype is computed only when ${\hat{s}}_{c}^{t}\geq s_{\min}$:

(44) $${\tilde{\mu }}_{c}^{t+1}=\frac{{\hat{a}}_{c}^{t}/\max\left({\hat{s}}_{c}^{t},s_{\min}\right)}{{∥{\hat{a}}_{c}^{t}/\max\left({\hat{s}}_{c}^{t},s_{\min}\right)∥}_{2}+\upsilon }.$$

The candidate is combined with the preceding protected prototype using a support-dependent coefficient:

(45) $$\begin{array}{cc} \alpha_{c}^{t} & ={\mathrm{clip}}_{\left[\alpha_{\min},\alpha_{\max}\right]}\;\left(\frac{\max({\hat{s}}_{c}^{t},0)}{\max\left({\hat{s}}_{c}^{t},0\right)+\tau_{s}}\right),\\ {\hat{\bar{\mu }}}_{c}^{t+1} & =\frac{\left(1-\alpha_{c}^{t}\right){\hat{\bar{\mu }}}_{c}^{t}+\alpha_{c}^{t}{\tilde{\mu }}_{c}^{t+1}}{{∥\left(1-\alpha_{c}^{t}\right){\hat{\bar{\mu }}}_{c}^{t}+\alpha_{c}^{t}{\tilde{\mu }}_{c}^{t+1}∥}_{2}+\upsilon }. \end{array}$$

If a class does not reach $s_{\min}$, its previous prototype is retained; if none exists, the class remains outside $G_{t+1}$. All quantities used to choose $M_{t+1}$ or form ${\hat{P}}^{t+1}$ are functions of protected aggregates. Importance scoring, support selection, normalization, and temporal smoothing therefore preserve the client-level guarantee in Equation (30).

### 4.4. End-to-End Training Procedure and Theoretical Analysis

The complete procedure integrates the preceding components into one privacy-accounted process. The server maintains $\theta^{t}$, ${\hat{P}}^{t}$, clipping thresholds, cumulative Rényi cost, coordinate-importance statistics, and staleness counters. A selected client maintains only its local artistic images and temporary optimization variables. Algorithm 1 summarizes training.
**Algorithm 1** FedArtSense Training in a Visual IoT Sensor Network**Input:** Clients K, rounds *T*, local epochs *E*, model $\theta^{0}$, target (ϵ,δ), minimum survivors $M_{\min}$, and nominal ratio $\gamma_{0}$.**Output:** Protected global model $\theta^{T}$ and prototype set ${\hat{P}}^{T}$. Initialize ${\hat{P}}^{0}\leftarrow ⌀$, clipping thresholds, importance statistics, staleness counters, and $\rho_{\mathrm{cum}}\left(\lambda \right)\leftarrow 0$.**for** t=0,…,T−1 **do**   Select $S_{t}$ and set $r_{t}=\left|S_{t}\right|/K$.   Construct $P^{t}$ from a public seed at t=0 or from protected importance and staleness statistics otherwise.   Allocate $\rho_{\mathrm{tar}}^{t}\left(\lambda \right)$ and determine $\sigma_{\Delta }^{t}$, $\sigma_{P}^{t}$, and $\sigma_{q}^{t}$.   Broadcast $\theta^{t}$, ${\hat{P}}^{t}$, $P^{t}$, clipping thresholds, and noise multipliers.   **for each** $k\in S_{t}$ in parallel **do**     Compute $d_{k}^{t}$ and $\beta_{k}^{t}$; perform $E_{k}^{t}$ local updates using Equation (17).     Compute $\Delta_{k}^{t}$ and ${\left\{\mu_{k,c}^{t}\right\}}_{c\in C_{k}}$; clip and protect all contributions.   **end for**   **if** at least $M_{\min}$ clients complete **then**     Securely aggregate messages; update $\theta^{t+1}$, ${\hat{P}}^{t+1}$, thresholds, and $\rho_{\mathrm{cum}}\left(\lambda \right)$.   **else**     Abort without releasing an aggregate or increasing the accountant.   **end if****end for**Convert $\rho_{\mathrm{cum}}\left(\lambda \right)$ to (ϵ,δ) and return $\theta^{T}$ and ${\hat{P}}^{T}$.

The initialization of $M_{0}$ is independent of private client data. After warm-up, all sparse supports are constructed from protected aggregates. A failed round releases no model, prototype, clipping statistic, or importance value and therefore consumes no additional privacy budget. When the privacy filter determines that another release would exceed the target, training terminates at the latest valid model.

**Optimization assumptions.** Let $J_{k}^{t}\left(\theta \right)$ denote the expected local objective in Equation (16) without the proximal term, and define

(46) $$J^{t}\left(\theta \right)=\sum_{k=1}^{K}p_{k}J_{k}^{t}\left(\theta \right).$$

The proximal term is excluded because it is centered at $\theta^{t}$ and is used only to constrain local drift. Every $J_{k}^{t}$ is assumed to be *L*-smooth and lower bounded by $J_{\inf}$. For a stochastic mini-batch gradient $g_{k}^{t}$, its conditional variance satisfies

(47) $$E\;\left[{∥g_{k}^{t}-\nabla J_{k}^{t}\left(\theta \right)∥}_{2}^{2}\right]\leq \sigma_{g}^{2}.$$

Client heterogeneity is bounded by $\zeta^{2}$:

(48) $$\sum_{k=1}^{K}p_{k}{∥\nabla J_{k}^{t}\left(\theta \right)-\nabla J^{t}\left(\theta \right)∥}_{2}^{2}\leq \zeta^{2}.$$

The temporal change of the protected prototype set makes $J^{t}$ round-dependent. Define prototype drift as

(49) $$D_{P}^{t}={\left(\sum_{c\in G_{t}\cap G_{t+1}}{∥{\hat{\bar{\mu }}}_{c}^{t+1}-{\hat{\bar{\mu }}}_{c}^{t}∥}_{2}^{2}\right)}^{1/2}.$$

We assume the induced gradient variation is bounded by

(50) $${∥\nabla J^{t+1}\left(\theta \right)-\nabla J^{t}\left(\theta \right)∥}_{2}\leq L_{P}D_{P}^{t}.$$

The shared projection is assumed to retain a nonzero fraction of global descent energy. For some ω∈(0,1],

(51) $${∥P^{t}\nabla J^{t}\left(\theta^{t}\right)∥}_{2}^{2}\geq \omega {∥\nabla J^{t}\left(\theta^{t}\right)∥}_{2}^{2}.$$

This condition does not require every coordinate to be selected in every round. The staleness component prevents indefinite exclusion and increases the effective ω over successive windows. The dense case gives ω=1.

Clipping introduces bias when a client contribution exceeds $R_{\Delta }^{t}$. Let

(52) $$b_{t}={∥E\;\left[\sum_{k\in S_{t}}u_{k,\Delta }^{t}-\sum_{k\in S_{t}}p_{k,t}\Delta_{k}^{t}\right]∥}_{2}.$$

The expected squared norm of aggregated model noise is bounded by

(53) $$V_{\mathrm{DP}}^{t}=\frac{M_{t}}{M_{\min}}m_{t}{\left(\sigma_{\Delta }^{t}R_{\Delta }^{t}\right)}^{2}.$$

**Convergence result.** Let $\eta_{\mathrm{eff}}=\eta_{s}\eta E$ be the effective round-level step size when every selected client performs *E* local epochs. Suppose $\eta_{\mathrm{eff}}\leq \omega /\left(4L\right)$ and the preceding assumptions hold. Then constants $C_{1},C_{2},C_{3}>0$, independent of *T*, exist such that

(54) $$\begin{array}{cc} \frac{1}{T}\sum_{t=0}^{T-1}E\;\left[{∥\nabla J^{t}\left(\theta^{t}\right)∥}_{2}^{2}\right] & \leq \frac{2(J^{0}\left(\theta^{0}\right)-J_{\inf})}{\omega \eta_{\mathrm{eff}}T}\\ \; & \;+\frac{C_{1}L\eta_{\mathrm{eff}}}{\omega }\left(\frac{\sigma_{g}^{2}}{\bar{M}}+E^{2}\zeta^{2}\right)\\ \; & \;+\frac{C_{2}}{\omega T}\sum_{t=0}^{T-1}\left[b_{t}^{2}+L_{P}^{2}{\left(D_{P}^{t}\right)}^{2}\right]\\ \; & \;+\frac{C_{3}L\eta_{s}^{2}}{\omega \eta_{\mathrm{eff}}T}\sum_{t=0}^{T-1}V_{\mathrm{DP}}^{t}, \end{array}$$

where $\bar{M}$ is the harmonic mean of the numbers of clients completing valid rounds.

The first term decreases as O(1/T). The second represents stochastic-gradient variance and non-IID client drift. Prototype-guided alignment and proximal regularization reduce the practical magnitude of $\zeta^{2}$, although the bound does not assume that heterogeneity vanishes. The third captures clipping bias and temporal variation in protected prototypes. Temporal smoothing directly limits $D_{P}^{t}$. The final term quantifies the cost of differential-privacy noise and exposes its dependence on $m_{t}$, $R_{\Delta }^{t}$, and $\sigma_{\Delta }^{t}$. Sparsification can therefore reduce communication and injected noise energy, provided that selected coordinates preserve sufficient descent energy.

The result follows by applying the smoothness inequality to Equation (42), decomposing the protected aggregate into its expected descent direction, client-sampling error, clipping bias, and Gaussian perturbation, and summing the one-round inequalities. Equation (51) lower-bounds retained descent by $\omega ∥\nabla J^{t}\left(\theta^{t}\right){∥}_{2}^{2}$. Young’s inequality separates clipping and prototype-drift cross terms, while independence and zero mean remove the first-order Gaussian term. A constant step size therefore converges to a stationary neighborhood determined by heterogeneity, clipping, prototype perturbation, and privacy noise.

**Computational and communication complexity.** Let $C_{f}$ denote the cost of one forward–backward pass. Client *k* requires $O(E_{k}^{t}n_{k}C_{f})$ operations for local learning and $O(n_{k}d_{z})$ to construct final prototypes. Clipping and projection require O(d) operations but do not require transmitting a dense vector. Client memory is $O(d+Cd_{z})$. The uplink message contains $m_{t}$ protected model values and $C(d_{z}+1)$ protected prototype and support values, giving $O(m_{t}+Cd_{z})$ values rather than O(d). At the server, importance and staleness updates cost O(d) per round; top-coordinate selection can be implemented in expected O(d) time or $O(d\log m_{t})$ time with a bounded heap. Secure aggregation requires $O(M_{t}\left(m_{t}+Cd_{z}\right))$ vector arithmetic per round and $O(M_{t}^{2})$ small control messages for the complete pairwise graph; Equation (40) gives the concrete $M_{t}=10$ accounting used for analytical reporting.

## 5. Experiments

### 5.1. Experimental Setup

We evaluate FedArtSense on three existing artistic image collections that differ in historical coverage, class balance, visual medium, and scale. WikiArt provides historical artworks with artist, genre, and style annotations; we use its 27-class style labels. The processed version is available at https://huggingface.co/datasets/huggan/wikiart (accessed on 20 May 2026), and task construction follows the refined WikiArt benchmark [14]. ArtBench-10 is a standardized, class-balanced collection containing ten artistic styles; it is available at https://github.com/liaopeiyuan/artbench (accessed on 20 May 2026) [15]. BAM contains contemporary artworks annotated with content, emotion, and artistic-medium attributes [4]. For BAM, we retain images with one dominant label among 3D computer graphics, comics, oil painting, pen-and-ink, pencil sketch, vector art, and watercolor, then sample 14,000 images per category to create a balanced seven-class subset of 98,000 images. Table 1 reports the resulting splits.

The original ArtBench-10 test set is retained, while 5000 images from its original training set are reserved for validation. WikiArt and BAM use stratified 70%/10%/20% train, validation, and test splits. Images belonging to the same source record are restricted to one split. The centralized validation sets are used only for hyperparameter selection; no validation image participates in federated optimization. All final metrics are calculated on held-out test sets.

To simulate visual IoT deployments, the training split of each dataset is distributed among K=50 clients. Client label proportions are sampled from a symmetric Dirichlet distribution with concentration α. A smaller α produces stronger label skew; the default is α=0.3, while α∈{0.1,0.3,0.5,1.0} is used in heterogeneity analysis. Resampling continues until every client receives at least 100 images and two labels. The centralized test set remains unchanged.

Each client is also assigned a fixed acquisition profile to approximate images collected by different cameras. The profile combines brightness scaling in [0.75,1.25], contrast scaling in [0.8,1.2], Gaussian blur with standard deviation in [0,1.2], and JPEG quality in [55,100]. Parameters are sampled once and then held constant per client, producing persistent sensor-domain shift without changing class labels. Training images are resized to 256×256 pixels, randomly cropped to 224×224, and horizontally flipped with probability 0.5. Validation and test images use deterministic resizing and center cropping.

Table 2 separates the deployment interpretation from the benchmark preprocessing. The public datasets do not contain continuous museum-camera streams; the listed acquisition frequency and source resolution define the visual IoT scenario used to interpret compute and communication feasibility.

**Figure 3:**
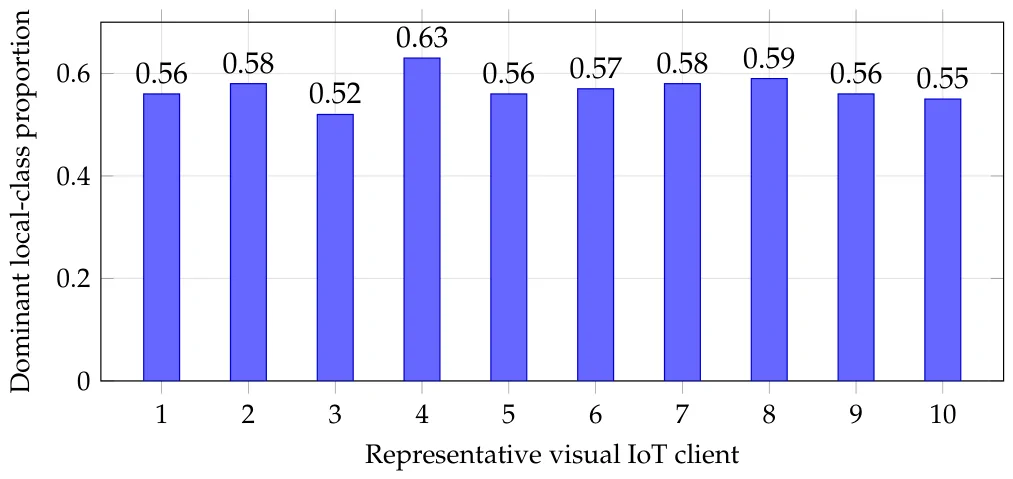
Dominant local-class proportions for ten representative ArtBench-10 clients at α=0.3. Clients 1–10 are respectively dominated by style indices 0–9: Art Nouveau, Baroque, Expressionism, Impressionism, Post-Impressionism, Realism, Renaissance, Romanticism, Surrealism, and Ukiyo-e.

Figure 3 illustrates one ArtBench-10 allocation at α=0.3. Although ten representative clients are shown for readability, the same process is applied to all 50 clients. Each client is dominated by a different artistic style, and the dominant share exceeds one half for most clients, illustrating strong label skew.

All methods use an ImageNet-pretrained ResNet-18 encoder with a 512-dimensional representation and a dataset-specific linear head [41]. The same initialization is shared among methods within each repetition. Local models use stochastic gradient descent with momentum 0.9, batch size 32, weight decay $5\times 10^{-4}$, and an initial learning rate of 0.01 followed by cosine decay. The server learning rate is $\eta_{s}=1$. Unless stated otherwise, ten clients participate in each of T=200 rounds, giving $r_{t}=0.2$, and each selected client performs E=3 local epochs.

To represent heterogeneous edge resources, clients are divided into low-, medium-, and high-capacity groups containing 30%, 50%, and 20% of clients. They perform one, three, and five local epochs, respectively, in the device-heterogeneity experiment, with nominal uplink rates of 0.8, 3, and 8 Mbit/s. Client selection remains uniform. Training is simulated in PyTorch v2.12.1 using four NVIDIA A100 GPUs; communication latency and transmitted bytes are calculated from the assigned profiles. Peak client memory and inference latency are additionally profiled on an NVIDIA Jetson Orin Nano. The Bonawitz control plane is analytically simulated from the packet model in Equation (40); its latency is not a measurement from a deployed cryptographic stack.

Table 3 lists the default FedArtSense configuration. Prototype parameters are selected using the ArtBench-10 validation set and fixed for the other datasets. The default client-level target is $\left(\epsilon ,\delta \right)=\left(6,10^{-5}\right)$, and additional experiments use ϵ∈{2,4,6,8}. The accountant evaluates λ∈{1.25,1.5,2,3,4,8,16,32,64}. A round is released only when at least $M_{\min}=8$ clients complete secure aggregation.

**Figure 4:**
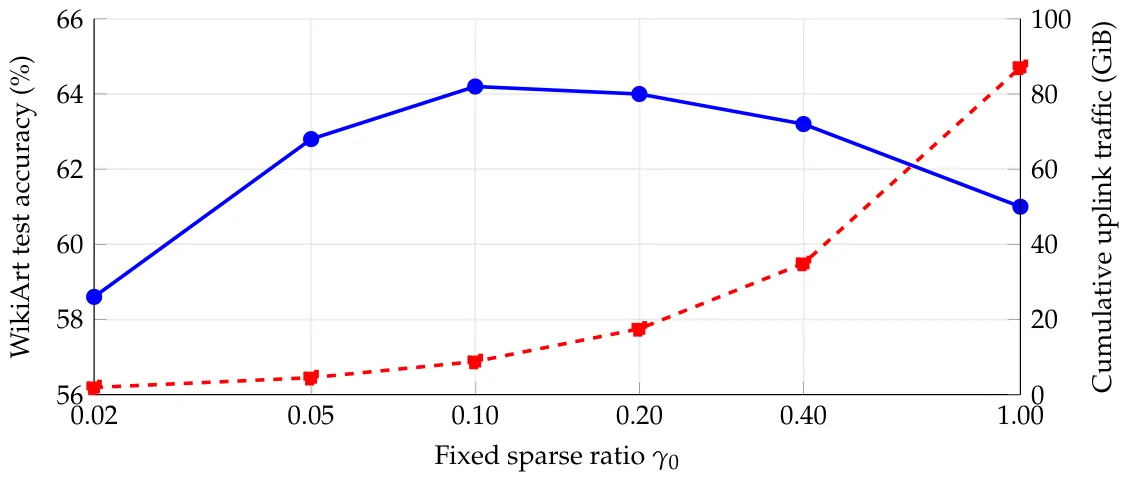
Effect of a fixed sparse ratio on WikiArt. The solid blue curve with circular markers, read from the left axis, shows accuracy. The dashed red curve with square markers, read from the right axis, shows cumulative traffic.

A practical tuning order reduces the apparent parameter count. First, deployment policy fixes (ϵ,δ), $M_{\min}$, and the communication budget. Second, Equation (37) determines a feasible $\gamma_{0}$; Figure 4 indicates that overly small ratios discard descent energy, whereas dense updates add traffic and privacy noise. Third, $q^{*}=0.8$ and $\kappa_{R}\in \left[0.1,0.2\right]$ are stable defaults when the desired clipped-client rate is near 20%, as reflected in Table 4. Finally, tune $\beta_{\max}$ and ξ from observed representation discrepancy, then χ, *m*, and τ on a validation set; $\lambda_{\mathrm{prox}}$ is increased only when local drift remains large. The component effects in Table 5 provide the priority order: prototype guidance and adaptive privacy are more sensitive than smoothing or exploration.

The exploration fraction has a limited two-point sensitivity result in Table 5. Setting $\gamma_{\exp}=0$ reduces WikiArt accuracy from 64.2% to 63.3% and ArtBench-10 accuracy from 81.2% to 80.6%; the default 0.10 prevents long-term coordinate starvation. Values in [0.05,0.20] are recommended when bandwidth permits, while larger values increasingly replace historically important coordinates. A broader sweep is not used to support the reported claims.

**Figure 5:**
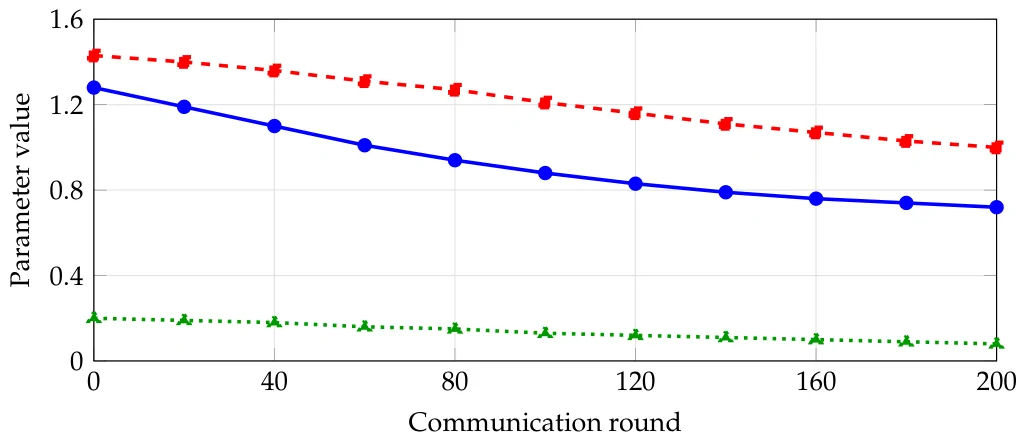
Adaptive profile in a representative WikiArt run. The solid blue curve with circular markers is $R_{\Delta }^{t}$, the dashed red curve with square markers is $\sigma_{\Delta }^{t}$, and the dotted green curve with triangular markers is $\gamma_{t}$.

The adaptive variables remain bounded throughout training. Figure 5 shows their trajectories for a representative WikiArt run with ϵ=6 and α=0.3. The model-update threshold decreases after warm-up, the multiplier decreases as more precision is allocated to refinement, and the sparse ratio retains more coordinates early before approaching its lower operating range.

The primary predictive metrics are top-1 accuracy and macro-F1. Privacy is reported using final client-level ϵ at $\delta =10^{-5}$. Communication is cumulative client-to-server traffic, and convergence is the number of rounds needed to reach 95% of a method’s final validation accuracy. Privacy leakage is examined using client-level membership-inference attack AUC. Every experiment is repeated with five partition and initialization seeds; tables report mean and standard deviation, and statistical comparisons use paired two-sided tests.

### 5.2. Comparison with Federated Learning Baselines

We compare FedArtSense with four non-private methods and three client-level private methods. FedAvg performs weighted averaging [1]; FedProx adds a proximal constraint [5]; SCAFFOLD uses control variates [6]; and MOON introduces model-level contrastive regularization [7]. DP-FedAvg and DP-FedProx apply client clipping and Gaussian perturbation using the same accountant and privacy target as FedArtSense [2]. Fed-SMP uses top-*m* sparsified model perturbation under client-level privacy [9]. All methods use the same initialization, client partitions, participation sequences, transformations, local optimizer, and 200-round limit. Non-private baselines exchange dense 32-bit updates; SCAFFOLD also communicates its control variate. Private methods are evaluated at $\left(\epsilon ,\delta \right)=\left(6,10^{-5}\right)$.

Table 6 presents test accuracy and macro-F1. FedArtSense achieves the best result among private methods on every dataset. Relative to Fed-SMP, it improves accuracy by 4.8, 4.4, and 4.1 percentage points on WikiArt, ArtBench-10, and BAM, respectively, with macro-F1 improvements of 5.0, 4.6, and 4.2 points.

Despite client-level differential privacy, FedArtSense exceeds non-private FedAvg on all datasets and approaches non-private MOON, with accuracy differences of 0.9, 0.4, and 0.6 points. This does not imply that privacy perturbation improves an identical optimizer; dual-space prototype guidance and heterogeneity-aware alignment compensate for part of the utility loss that conventional private averaging experiences on strongly non-IID artistic images.

**Figure 6:**
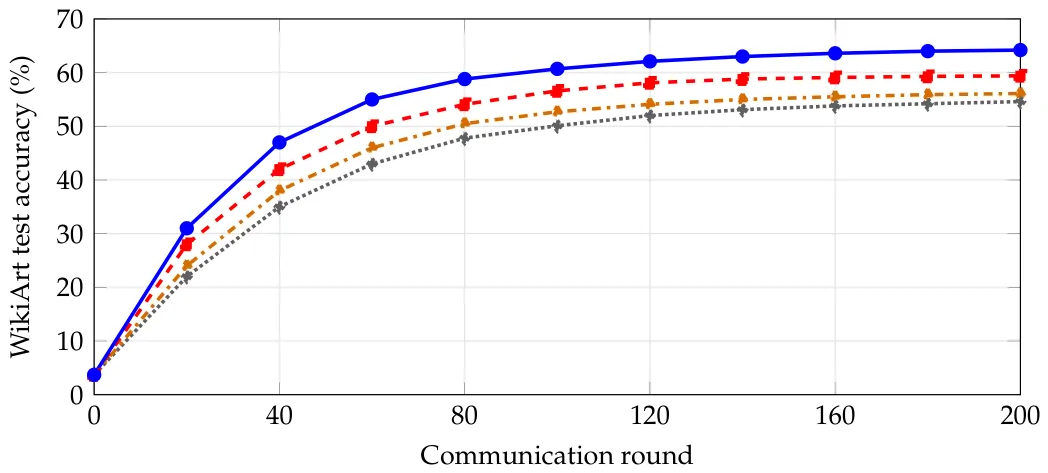
Convergence of private methods on WikiArt. The gray dotted curve with diamond markers is DP-FedAvg, the orange dash-dotted curve with triangular markers is DP-FedProx, the red dashed curve with square markers is Fed-SMP, and the solid blue curve with circular markers is FedArtSense.

The WikiArt convergence curves in Figure 6 show that the advantage develops throughout training. DP-FedAvg and DP-FedProx oscillate after round 80 because fixed clipping and noise do not follow the declining scale of client updates. Fed-SMP reduces perturbation dimension, but its sparse updates do not explicitly address representation drift. FedArtSense reaches 60% accuracy near round 94 and improves to 64.2%.

Table 7 compares privacy leakage, communication, and convergence on WikiArt. Communication uses actual shared-support encoding, in which the sparse coordinate order is broadcast once per round. FedArtSense reduces uplink traffic by 86.8% relative to dense FedAvg and 24.3% relative to Fed-SMP. Its prototype payload contributes 0.18 GiB and is included.

The client-level attack trains a binary classifier from model-loss statistics, prediction entropy, and confidence changes measured on complete client collections. Non-private methods yield AUC values between 0.681 and 0.712, showing that keeping images local is insufficient to prevent participation inference. Private methods reduce the attack close to random guessing. FedArtSense obtains 0.508, while its accountant reports ϵ=5.98, slightly below target because the implementation searches discrete noise-multiplier and Rényi-order grids.

The 11.5 GiB value in Table 7 includes sparse model values, shared-support encoding, and protected prototypes, but excludes secure-aggregation control packets. With $M_{t}=10$ and $M_{\min}=8$, Equation (40) gives 2.55 KiB of bidirectional control traffic per client per released round, of which 1.18 KiB is uplink. Across 200 released rounds, this adds 2.31 MiB to cumulative uplink and 4.99 MiB bidirectionally; including control uplink changes 11.5 GiB to 11.502 GiB. Table 8 reports the corresponding analytical latency under the three network tiers.

**Figure 7:**
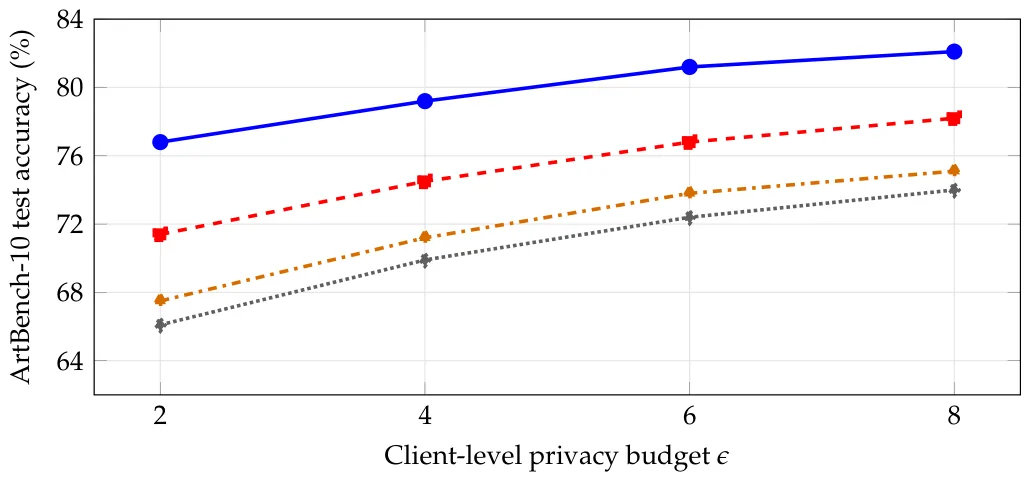
Privacy–utility comparison on ArtBench-10 at $\delta =10^{-5}$. The gray dotted curve with diamond markers is DP-FedAvg, the orange dash–dot curve with triangular markers is DP-FedProx, the red dashed curve with square markers is Fed-SMP, and the solid blue curve with circular markers is FedArtSense.

Figure 7 examines the privacy–utility trade-off on ArtBench-10. Accuracy increases with ϵ because a larger budget permits weaker perturbation. Relative to Fed-SMP, FedArtSense improves accuracy by 5.4 points at ϵ=2 and 3.9 points at ϵ=8, indicating that prototype alignment and adaptive coordinate selection are particularly useful under strong perturbation.

Paired comparisons across five repetitions show that FedArtSense significantly outperforms Fed-SMP in accuracy on all three datasets, with p<0.01 in each case. Agreement among predictive performance, convergence speed, communication volume, and attack AUC indicates that improvement is not obtained by relaxing privacy or increasing communication. Figure 7 also clarifies that the default ϵ=6 is a moderate, not high-assurance, client-level guarantee. Reducing the budget to ϵ=2 lowers ArtBench-10 accuracy from 81.2% to 76.8%, a 4.4-point decrease. Budgets below 2, including ϵ≤1, are not evaluated and are not covered by the deployment claim.

### 5.3. Ablation and Sensitivity Analysis

We conduct component-wise ablations to determine whether the performance gain arises from one mechanism or from the interaction among representation alignment, adaptive privacy, and communication-aware aggregation. Each ablation removes one component while retaining the same partitions, privacy target, communication rounds, and optimizer. Table 5 reports results on WikiArt and ArtBench-10.

Removing prototype guidance sets $\beta_{k}^{t}=0$ and produces the largest decrease, reducing WikiArt accuracy to 59.8%. Retaining feature alignment but setting χ=0 lowers accuracy by 1.5 points, showing that consistency between prototype geometry and the classification head contributes beyond feature attraction. Replacing $\beta_{k}^{t}$ with constant $\beta_{\max}$ also reduces performance because well-aligned clients become over-regularized. Replacing adaptive privacy with a fixed threshold and uniform expenditure loses 3.7 points. A public random support of the same size loses 2.6 points, showing that the gain does not arise from sparsity alone.

**Figure 8:**
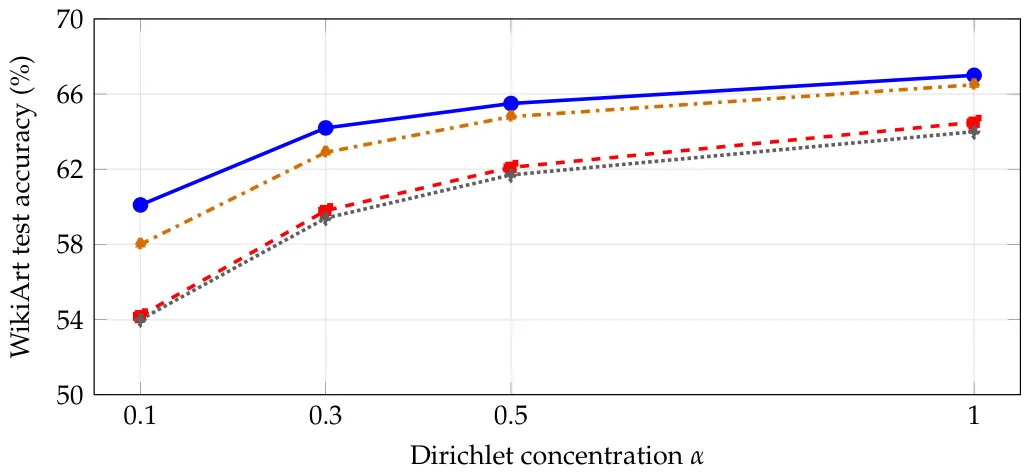
Sensitivity to label heterogeneity on WikiArt. The solid blue curve with circular markers is FedArtSense, the red dashed curve with square markers removes prototype guidance, the orange dash–dot curve with triangular markers uses a fixed alignment coefficient, and the gray dotted curve with diamond markers is Fed-SMP.

The effect of prototype guidance becomes more pronounced as client distributions become more heterogeneous. Figure 8 compares the complete method with its principal representation ablations and Fed-SMP over four Dirichlet concentrations. At α=0.1, removing prototype guidance reduces accuracy by 5.9 points, compared with 2.5 points at α=1.0.

To separate adaptive privacy operations, Table 4 compares fixed and adaptive clipping with uniform and progress-aware allocation. All variants satisfy approximately the same final budget. The clipped-client rate is the proportion of client updates whose weighted norm exceeds $R_{\Delta }^{t}$. Normalized noise energy is calculated from $\sum_{t}V_{\mathrm{DP}}^{t}$ and divided by the fixed-clipping, uniform-allocation value.

Adaptive clipping alone reduces noise energy to 0.82 because its threshold follows the declining update scale. Progress-aware allocation alone reduces energy to 0.87 by preserving privacy resources for late refinement. Their combination yields the lowest noise energy and highest accuracy. The combined clipped-client rate remains close to the intended $1-q^{*}=20\%$, confirming that private quantile feedback tracks its target.

We next hold the sparse ratio constant by setting $\gamma_{\min}=\gamma_{\max}=\gamma_{0}$. Figure 4 reports WikiArt accuracy and cumulative traffic. Extremely aggressive compression at $\gamma_{0}=0.02$ loses important descent coordinates. Increasing the ratio to 0.10 improves accuracy; beyond this point, extra coordinates increase communication and the norm of Gaussian perturbation, causing a gradual decrease under the fixed privacy budget.

The best fixed ratio is $\gamma_{0}=0.10$, which is used as the center of the adaptive interval. The adaptive policy communicates more than the fixed-0.10 variant because it retains extra coordinates during early high-progress rounds, but it reaches 95% of final accuracy 18 rounds earlier.

### 5.4. Robustness and Edge-Deployment Evaluation

Visual IoT sensors can introduce persistent appearance changes absent from curated benchmarks. We evaluate four acquisition-shift levels. No shift uses deterministic resizing and cropping. Mild shift uses brightness and contrast factors in [0.9,1.1], blur up to 0.4, and JPEG quality in [75,100]. Moderate shift is the default profile in Section 5.1. Severe shift expands brightness to [0.6,1.4], contrast to [0.65,1.35], maximum blur standard deviation to 2.0, and JPEG quality to [35,85]. Each client retains one profile. Table 9 reports accuracy on WikiArt and BAM.

Acquisition shift reduces every method’s performance, but FedArtSense remains most accurate at each severity. Under severe shift, it exceeds Fed-SMP by 5.8 points on WikiArt and 4.6 on BAM. Its decrease from no shift to severe shift is 4.1 points on both datasets, compared with 5.6 and 5.1 for DP-FedAvg. Protected prototypes provide a semantic reference less sensitive to client-specific brightness, compression, and blur than direct parameter averaging.

**Figure 9:**
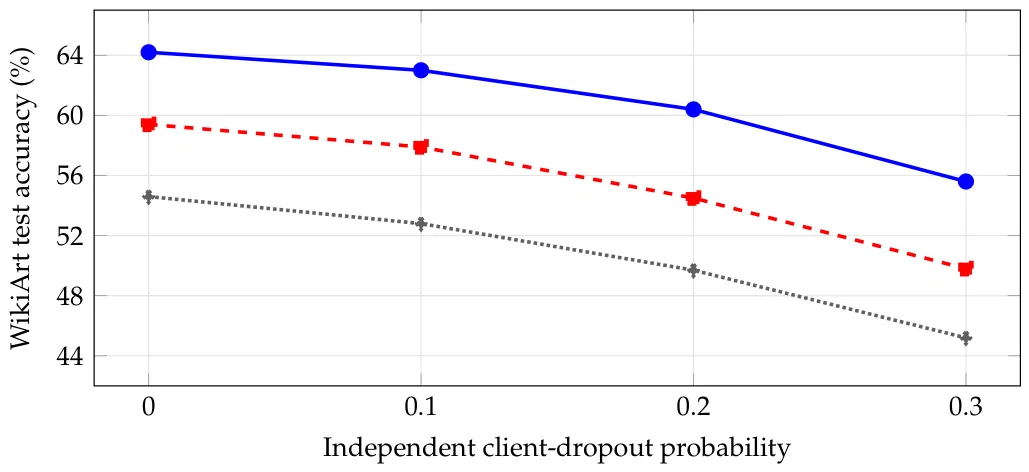
Robustness to client dropout over 200 attempted rounds. The gray dotted curve with diamond markers is DP-FedAvg, the red dashed curve with square markers is Fed-SMP, and the solid blue curve with circular markers is FedArtSense.

We simulate independent client dropout after local training but before secure aggregation. The server attempts 200 rounds, and an attempted round is released only if at least $M_{\min}=8$ of ten selected clients remain. Aborted rounds reveal no aggregate and consume no privacy budget. Figure 9 shows WikiArt accuracy as dropout increases. FedArtSense remains above 60% at dropout probability 0.2, whereas Fed-SMP and DP-FedAvg decrease to 54.5% and 49.7%.

The advantage under dropout has two sources. Temporally smoothed prototypes preserve class information from earlier valid rounds when a later contribution is unavailable, and importance scores use the most recent released aggregate rather than attempted rounds. At dropout probability 0.3, only 78.4±4.1 attempted rounds satisfy the release threshold, so the remaining decrease primarily reflects fewer optimization steps.

We profile client execution on an NVIDIA Jetson Orin Nano using batch size 32 and one local epoch over 500 WikiArt images. Table 10 reports training time, peak memory, relative energy, and average uplink payload. Inference latency is measured for one 224×224 image after ten warm-up iterations and averaged over 1000 runs.

Prototype extraction and dual-space alignment increase local-epoch time by 11.8% and peak memory by 0.22 GiB relative to DP-FedAvg. This overhead is offset by an 86.8% reduction in average uplink payload. FedArtSense also sends 24.4% fewer bytes than Fed-SMP because its coordinate support is shared. The 0.1 ms inference difference lies within measurement variation, showing that training does not increase deployment-time model complexity.

Table 10 supports gateway-class deployment, not direct training on a battery-powered camera. MobileNetV3 and EfficientNet provide architecture families designed for resource-constrained inference and training [42,43], but lightweight backbones and quantization-aware training are not evaluated here. FedArtSense is architecturally compatible with them because its model payload scales with parameter dimension *d* and its prototype payload with representation dimension $d_{z}$; their accuracy, clipping, and privacy schedules require separate calibration. The reported 161.9 s local epoch and 2.93 GiB peak memory therefore define the ResNet-18 gateway profile rather than a battery-sensor requirement.

**Figure 10:**
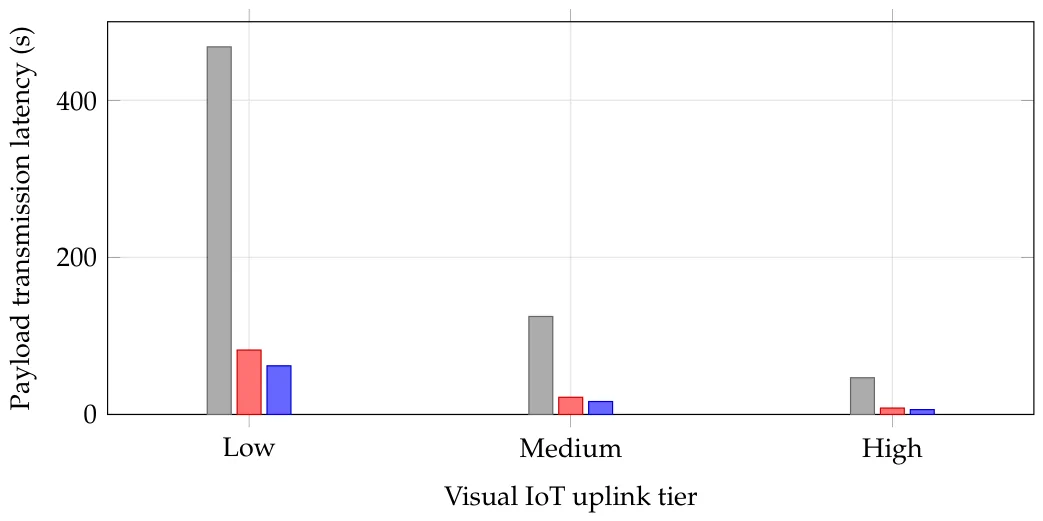
Estimated masked-payload transmission latency for one client message, excluding the control-plane values reported in Table 8. Within each tier, the left gray bar represents DP-FedAvg, the middle red bar Fed-SMP, and the right blue bar FedArtSense. Low-, medium-, and high-tier rates are 0.8, 3, and 8 Mbit/s, respectively.

Figure 10 translates only the masked model/prototype payloads into transmission latency for the bandwidth tiers in Section 5.1; Table 8 separately reports the Bonawitz control plane. At 0.8 Mbit/s, a dense DP-FedAvg update requires approximately 468 s, compared with 82 s for Fed-SMP and 62 s for FedArtSense. The additional FedArtSense control latency is 0.776 s under the low-tier RTT assumption.

These results indicate suitability for bandwidth-constrained, gateway-assisted visual IoT deployments when moderate additional local computation is available. The main deployment limitation is that secure aggregation requires at least $M_{\min}$ surviving clients. Networks with frequent correlated outages would require a smaller release threshold with recalibrated noise or a larger initially selected set.

### 5.5. Limitations and Practical Scope

The evaluation is limited to style and medium classification on curated public datasets with simulated client partitions and acquisition transformations. Table 2 defines the intended camera–gateway scenario, and Table 10 shows that the current ResNet-18 training profile is unsuitable for many battery-powered or thermally constrained sensors. Lightweight backbones, quantization-aware training, live museum streams, retrieval, aesthetic prediction, and detection remain unevaluated.

The default ϵ=6 setting is moderate. Figure 7 reports stronger protection at ϵ=2, but no result is available for ϵ<2. Differential privacy protects one client’s participation in the complete transcript; it does not hide aggregate semantic information shared by many institutions. Consequently, the protected prototype sequence may reveal federation-level prevalence of styles or media, and a dedicated prototype-attribute inference evaluation remains open.

Encrypted image exchange and reversible perturbation provide complementary content protection. End-to-end encryption can be preferable when an institution accepts an authorized central analyst and needs confidentiality in transit, but it scales with image volume and exposes plaintext at the authorized endpoint [34]. Reversible perturbation can protect locally stored or intercepted frames independently of the federated transcript, but adversarial distortion may alter local features and prototype geometry, while recovery keys add another trust boundary [35]. Applying such a transform before local training can provide defense in depth, but it does not reduce the stated (ϵ,δ) budget unless the combined mechanism is analyzed, and its effect on dual-space alignment requires separate validation.

## 6. Conclusions

This paper presented FedArtSense for privacy-preserving artistic style and medium classification across distributed visual IoT gateways. It combines discrepancy-adaptive dual-space prototype alignment, adaptive client-level differential privacy, shared importance-aware sparsification, and Bonawitz-style secure aggregation. On WikiArt, ArtBench-10, and BAM, the method obtains 64.2%, 81.2%, and 75.3% accuracy under the default $\left(\epsilon ,\delta \right)=\left(6,10^{-5}\right)$ target. The WikiArt cumulative masked-payload uplink is 11.5 GiB, and the analytically accounted secure-aggregation control uplink adds 2.31 MiB over 200 released rounds. The conclusions are restricted to classification, gateway-class ResNet-18 execution, and the evaluated privacy range ϵ∈{2,4,6,8}. Future work will study ϵ<2, lightweight and quantized backbones, prototype-specific inference, live sensor streams, correlated failures, and non-classification art tasks.

## Figures and Tables

**Table 1:** Statistics of the artistic image datasets. WikiArt and ArtBench-10 use style classification; the filtered BAM subset uses medium classification.

Dataset	Task	Classes	Total Images	Training	Validation	Testing
WikiArt	Style classification	27	81,444	57,010	8145	16,289
ArtBench-10	Style classification	10	60,000	45,000	5000	10,000
BAM subset	Medium classification	7	98,000	68,600	9800	19,600

**Table 2:** Deployment assumptions used for the edge-feasibility analysis.

Item	Assumption
Visual source	Fixed RGB documentation/surveillance camera connected to an institutional gateway
Excluded sources	Visitor-owned phones, hyperspectral scanners, and direct on-camera training
Source/model resolution	1920×1080 or higher source frame; 256×256 resize and 224×224 crop
Acquisition schedule	Event-triggered or one frame every 5–30 s; local buffering before training
Local execution	Periodic gateway-assisted training; 200 rounds are not synchronized to camera frames
Connectivity	Camera–gateway Ethernet/Wi-Fi; gateway uplink tiers of 0.8/3/8 Mbit/s

**Table 3:** Default optimization, prototype, privacy, and sparsification hyperparameters.

Component	Hyperparameter	Default Value
Federated optimization	Clients/clients per round/rounds	50/10/200
	Local epochs/batch size	3/32
	Client learning rate/server learning rate	0.01/1.0
	SGD momentum/weight decay	$0.9/5\times 10^{-4}$
Prototype alignment	$\beta_{\max}$/ξ/χ	0.5/0.1/0.5
	Cosine margin *m*/temperature τ	0.2/0.1
	Proximal coefficient $\lambda_{\mathrm{prox}}$	$10^{-3}$
	Prototype smoothing $\left[\alpha_{\min},\alpha_{\max}\right]$	[0.1,0.8]
Privacy protection	Default (ϵ,δ)	$\left(6,10^{-5}\right)$
	Minimum surviving clients $M_{\min}$	8
	Target unclipped fraction $q^{*}$	0.8
	Threshold adaptation rate $\kappa_{R}$	0.15
	Privacy shares $\left(\pi_{\Delta },\pi_{P},\pi_{q}\right)$	(0.72,0.18,0.05) (fixed heuristic)
Sparse aggregation	$\gamma_{0}$/$\left[\gamma_{\min},\gamma_{\max}\right]$	0.10/[0.05,0.20]
	Exploration fraction $\gamma_{\exp}$	0.10
	Magnitude/update momentum	0.9/0.9
	Consistency/staleness weight	0.5/0.05

**Table 4:** Ablation of adaptive clipping and progress-aware privacy allocation on WikiArt. Fixed clipping with uniform allocation defines normalized noise energy 1.00.

Clipping	Allocation	Accuracy (%)	Macro-F1 (%)	Clipped Clients (%)	Noise Energy	Final ϵ
Fixed	Uniform	60.5±0.7	56.9±0.8	24.8±1.3	1.00	5.99
Adaptive	Uniform	62.1±0.6	58.4±0.7	19.6±1.1	0.82	5.99
Fixed	Progress-aware	61.7±0.6	58.1±0.7	24.3±1.2	0.87	5.98
Adaptive	Progress-aware	64.2±0.5	60.7±0.5	20.1±1.0	0.69	5.98

**Table 5:** Component-wise ablation under client-level $\left(6,10^{-5}\right)$-differential privacy. Each row removes one component; results are mean ± standard deviation over five repetitions.

Variant	WikiArt Accuracy (%)	WikiArt Macro-F1 (%)	ArtBench Accuracy (%)	ArtBench Macro-F1 (%)
FedArtSense	64.2±0.5	60.7±0.5	81.2±0.3	80.7±0.4
Without prototype guidance	59.8±0.6	56.0±0.7	77.0±0.5	76.4±0.5
Without decision-space alignment	62.7±0.5	59.1±0.6	79.9±0.4	79.4±0.4
Without heterogeneity-aware alignment	62.9±0.5	59.4±0.6	80.1±0.4	79.6±0.4
Without adaptive privacy control	60.5±0.7	56.9±0.8	77.8±0.5	77.2±0.6
Without importance scoring	61.6±0.6	58.0±0.7	78.9±0.5	78.3±0.5
$\gamma_{\exp}=0$	63.3±0.5	59.8±0.5	80.6±0.4	80.1±0.4
Without prototype smoothing	62.8±0.6	59.2±0.6	80.0±0.4	79.5±0.5

**Table 6:** Test accuracy and macro-F1 under the default non-IID partition. Private methods satisfy client-level $\left(6,10^{-5}\right)$-differential privacy. Results are mean ± standard deviation over five repetitions; bold indicates the best private result.

Method	DP	WikiArt		ArtBench-10		BAM Subset	
		Accuracy (%)	Macro-F1 (%)	Accuracy (%)	Macro-F1 (%)	Accuracy (%)	Macro-F1 (%)
FedAvg	No	61.8±0.6	58.4±0.7	79.1±0.4	78.7±0.5	73.5±0.5	71.6±0.6
FedProx	No	63.2±0.5	60.0±0.6	80.3±0.4	79.9±0.4	74.7±0.4	72.8±0.5
SCAFFOLD	No	64.0±0.5	60.8±0.5	80.7±0.3	80.3±0.4	75.1±0.4	73.4±0.4
MOON	No	65.1±0.4	62.1±0.5	81.6±0.3	81.2±0.3	75.9±0.3	74.3±0.4
DP-FedAvg	Yes	54.6±0.8	50.8±0.9	72.4±0.6	71.8±0.6	67.9±0.7	65.5±0.8
DP-FedProx	Yes	56.1±0.7	52.4±0.8	73.8±0.5	73.1±0.6	69.0±0.6	66.7±0.7
Fed-SMP	Yes	59.4±0.6	55.7±0.7	76.8±0.5	76.1±0.5	71.2±0.5	68.9±0.6
FedArtSense	Yes	64.2±0.5	60.7±0.5	81.2±0.3	80.7±0.4	75.3±0.4	73.1±0.4

**Table 7:** Privacy and efficiency on WikiArt. Attack AUC measures client-level membership inference; 0.5 represents random guessing. Rounds to 95% are relative to each method’s final validation accuracy.

Method	Final ϵ	Attack AUC	Uplink (GiB)	Rounds to 95%
FedAvg	–	0.712±0.014	87.1	149±6
FedProx	–	0.694±0.013	87.1	137±5
SCAFFOLD	–	0.701±0.012	174.2	118±5
MOON	–	0.681±0.011	87.1	124±4
DP-FedAvg	6.00	0.531±0.009	87.1	172±7
DP-FedProx	6.00	0.524±0.008	87.1	158±6
Fed-SMP	6.00	0.519±0.007	15.2	146±5
FedArtSense	5.98	0.508±0.006	11.5	121±4

**Table 8:** Analytical Bonawitz secure-aggregation control overhead for ten selected clients and an 8-of-10 recovery threshold. Transfer time uses 2.55 KiB/client; total latency adds three server-mediated round trips.

Tier	Rate (Mbit/s)	RTT Assumption (ms)	Transfer (s)	Control Latency (s)
Low	0.8	250	0.026	0.776
Medium	3	100	0.007	0.307
High	8	40	0.003	0.123

**Table 9:** Test accuracy under increasing client-specific sensor shift. All methods satisfy client-level $\left(6,10^{-5}\right)$-differential privacy.

Method	WikiArt Accuracy (%)				BAM Accuracy (%)			
	None	Mild	Moderate	Severe	None	Mild	Moderate	Severe
DP-FedAvg	56.8±0.7	55.8±0.7	54.6±0.8	51.2±0.9	69.4±0.6	68.8±0.6	67.9±0.7	64.3±0.8
DP-FedProx	58.2±0.6	57.4±0.7	56.1±0.7	52.9±0.8	70.3±0.5	69.8±0.6	69.0±0.6	65.7±0.7
Fed-SMP	61.1±0.6	60.4±0.6	59.4±0.6	55.8±0.8	72.6±0.5	72.0±0.5	71.2±0.5	67.9±0.7
FedArtSense	65.7±0.4	65.0±0.5	64.2±0.5	61.6±0.6	76.6±0.4	76.0±0.4	75.3±0.4	72.5±0.5

**Table 10:** Edge-device profile on an NVIDIA Jetson Orin Nano. Uplink includes sparse indices when required and protected prototypes for FedArtSense. Energy is normalized to DP-FedAvg.

Method	Local Epoch (s)	Peak Memory (GiB)	Relative Energy	Uplink/Client (MiB)	Inference (ms)
DP-FedAvg	144.8±1.9	2.71	1.00	46.8	18.6±0.3
DP-FedProx	148.2±2.0	2.74	1.02	46.8	18.6±0.3
Fed-SMP	152.6±2.1	2.78	1.05	8.2	18.6±0.3
FedArtSense	161.9±2.2	2.93	1.11	6.2	18.7±0.3

## Data Availability

The public datasets used in this study are available from the sources identified in Section 5.1.
